# Converge or Diverge? Exploring the Fate of Taxonomically Different Anaerobic Digestion Communities Under Uniform Growth Conditions

**DOI:** 10.1111/1751-7915.70233

**Published:** 2025-09-24

**Authors:** Vasiliki Tsamadou, Jonas A. Ohlsson, Anna Schnürer

**Affiliations:** ^1^ Department of Molecular Sciences, BioCenter, Box 7015 Swedish University of Agricultural Sciences Uppsala Sweden

**Keywords:** defined medium, determinism, inoculum source, microbial assembly, stochasticity, syntrophic acetate oxidation, total ammoniacal nitrogen

## Abstract

Biogas inocula with distinct taxonomic compositions often converge to similar communities when fed the same substrate, indicating strong substrate‐driven deterministic assembly. Nevertheless, stochastic processes have also been suggested as a critical element for microbial assembly in biogas systems. To date, assembly processes have mainly been investigated with undefined, non‐sterile substrates, making it difficult to exclude the influence of external microorganisms. The aim of the present study was to investigate whether three taxonomically distinct anaerobic digestion (AD) communities would converge when exposed to uniform growth conditions during semi‐continuous operation with a sterilised defined medium. The inocula originated from mesophilic processes using different substrates (food waste, sludge, and manure) and total ammonia levels (0.5–7.2 g/L). The medium was formulated to support all four main metabolic steps of AD: hydrolysis, fermentation, anaerobic oxidation, and methanogenesis. Taxonomic, phylogenetic, and functional analyses conducted via 16S and metagenomic sequencing showed that the substrate had no deterministic effect on microbial community taxonomic composition. Instead, the final community structure was dictated primarily by the initial inoculum, regardless of changes in substrate composition or ammonia levels. Despite taxonomic divergence, broad‐level functionality and operational performance remained similar between communities.

## Graphical abstract

Three semi‐continuous enrichments, inoculated with distinctly different microbial communities, were run for 3 HRTs using a defined nutrient medium supporting all anaerobic digestion steps. 16S and metagenomic analyses showed final community structures were driven primarily by the initial inoculum, while the substrate had no deterministic effect.

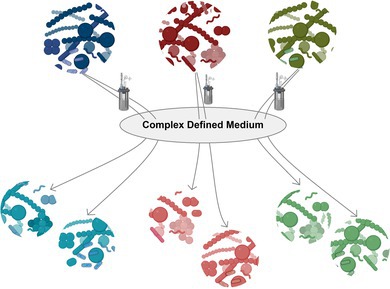

## 1 Introduction

Anaerobic digestion (AD) is a proven technology for converting organic waste into biogas and nutrient‐rich digestate, useful as fertiliser (Kougias and Angelidaki 2018). The process involves four main microbial steps: hydrolysis, fermentation, anaerobic oxidation and methanogenesis, each carried out by different microbial groups (Schnürer 2016). The first three steps are performed mainly by different bacterial taxa, while methanogenesis is performed exclusively by archaea. The bacterial community is comparably more diverse, both phylogenetically and functionally, while methanogens include three functional groups: acetoclastic (utilises acetate), hydrogenotrophic (utilises H_2_, CO_2_, formate) and methylotrophic (utilises methylated compounds like methanol or methylamines) (Enzmann et al. 2018). The assembly of the AD microbiome is mediated by parameters which can be categorised as either deterministic (e.g., abiotic environmental parameters, interspecies interactions) or stochastic (e.g., cell deaths and divisions, random dispersal of individual cells). In the environment, stochasticity and determinism have been shown to exist on a continuous spectrum, with various ecological factors modulating their relative contributions to community structure (Chase and Myers 2011; Yuan et al. 2019). In line with this, studies on various bioprocesses, including AD, have also highlighted both the importance of stochastic processes in shaping microbial communities (Ayarza and Erijman 2011; Zhou et al. 2013), as well as deterministic factors (Peces et al. 2018; Vanwonterghem et al. 2014). Factors known to strongly influence the microbial community structure in anaerobic digestion systems include process temperature, substrate characteristics and inoculum source (De Vrieze, Saunders, et al. 2015; Westerholm and Schnürer 2019). Substrate physicochemical characteristics have been shown to assert a deterministic effect on the composition of AD communities, both when using natural feedstocks (De Vrieze, Gildemyn, et al. 2015; Duan et al. 2021; Liu et al. 2017) and defined media (Peces et al. 2018). However, a few studies suggest that initial community structure, not type of substrate, is the main deciding factor for the makeup of the AD community (Han et al. 2016; Liu et al. 2018), highlighting stochasticity as a critical element for microbial community structure in biogas systems.

A specific aspect of substrate composition which has a strong deterministic effect on AD community makeup is its protein and ammonia (NH_3_) content. Ammonia, released during the degradation of proteins, is a well‐known inhibitor of AD processes and is associated with changes to the AD microbial community and loss of microbial diversity (De Vrieze, Saunders, et al. 2015; Li et al. 2015). Acetoclastic methanogens are known to be specifically inhibited by ammonia (Capson‐Tojo et al. 2020), while inhibition of acetate and propionate oxidation has also been reported (Bonk et al. 2018; Wang et al. 2015). However, functioning biogas‐producing systems with high ammonia concentrations can be achieved (Borja et al. 1996; Morozova et al. 2020). In these systems a microbial shift occurs, whereby acetoclastic methanogens are replaced by syntrophic acetate oxidizers and ammonia‐tolerant hydrogenotrophic methanogens (Müller et al. 2016; Westerholm et al. 2016).

As mentioned above, previous studies have not been able to reach a consensus on the relative influence of the initial microbial community and substrate characteristics on the structure of the microbial community in biogas processes, motivating further investigation. The aim of this study was to investigate whether taxonomically different biogas microbial communities would converge to similar taxonomy or not when cultivated under the same conditions using a sterile defined medium. For this, three AD microbial communities were selected to be as taxonomically distinct as possible. The inocula were taken from well‐functioning biogas processes based on complex feedstocks (manure, sludge and food waste), with different total ammoniacal nitrogen (TAN) concentrations. These cultures were axenically grown in lab‐scale bioreactors under either native (1, 2 and 7 g/L) or experimental (4 g/L) TAN concentrations. The changes in microbial community structure were monitored during 12 weeks in order to ascertain whether the change in TAN concentration and substrate composition would cause a convergence of the microbial communities.

## 2 Experimental Procedures

### 2.1 Experimental Design and Reactor Operation

Three pairs of laboratory‐scale continuous stirred‐tank reactors (Belach Bioteknik, Sweden) were used in this study. Each pair of reactors was inoculated with inoculum from different sources, which were selected to represent different categories of biogas processes: wastewater sludge (SL), manure (MN) and food waste (FW)‐based. These processes were separated not only by their substrate composition but also by different TAN levels: 0.5, 1.5 and 7 g/L, respectively. Inocula were taken from full‐scale processes except for FW, which was taken from an experimental reactor fed with food waste and extra albumin (reactor D^TE^37 from Westerholm et al. (2015)). All processes were operated at mesophilic temperature (37°C). Additional information on the inocula is presented in Table 1.

**TABLE 1:** Characteristics of biogas inocula used for initiation of bioreactors.

Name	Substrate	pH	TAN (g/L)	NH_3_ (g/L)	Acetate (g/L)	Total VFA (g/L)	TS (%)	VS (%)	COD (g/L)
SL	Wastewater sludge	7.22	0.51	0.01	0.04	0.04	3.95	2.66	10.50
MN	Manure and straw	7.87	1.48	0.13	0.03	0.24	4.51	3.18	46.40
FW	Food waste and albumin	8.02	7.17	0.85	2.44	3.87	2.11	1.15	23.80

*Note:* TAN, total ammoniacal nitrogen, COD, chemical oxygen demand; VFA, volatile fatty acid, also including: for MN 0.02 g/L lactate, 0.19 g/L propionate; for FW 0.04 g/L lactate, 0.88 g/L propionate, 0.11 g/L isobutyrate, 0.08 g/L butyrate, 0.25 g/L isovalerate and 0.06 g/L valerate. TS, total solids; VS, volatile solids, both expressed as a percentage of wet weight.

Each reactor (total volume 4 L) was filled with 1.5 L of inoculum while flushing with N_2_ gas. The SL and FW inocula were added intact, while the MN inoculum was passed through a 2 mm sieve to remove larger fibre fractions. Continuous feeding of the reactors with defined medium was initiated at inoculation. The reactors were operated at 37°C and a hydraulic retention time (HRT) of approximately 28 days, with gas bags attached for collection of the produced gas. Within each pair of reactors, one was being fed with medium with TAN levels that matched its inoculum source (reference reactor), while the other was fed with medium with 4 g/L TAN (experimental reactor). In this way, there were three experimental reactors (SL‐exp, MN‐exp, FW‐exp) and three reference reactors (SL‐ref, MN‐ref, FW‐ref). Thus, there was an increase in TAN in the SL and MN experimental reactors and a decrease in the FW experimental reactor. The total run time was approximately 3 HRT, approximately 3 months, to give a sufficient amount of time for the establishment and adjustment of the microbiological community to the new conditions.

### 2.2 Analytical Methods

Operational parameters were monitored weekly. pH was measured with a standard pH meter (Jenway 3510). Methane concentration in the reactor headspace was analysed using gas chromatography, as described previously (Westerholm et al. 2010). Total gas volume was measured every few days by emptying the gas bags with a Ritter Drum‐type Gas Meter (TG05/5).

Six volatile fatty acids (VFAs; acetic, propionic, butyric, isobutyric, valeric and isovaleric acid), and lactic acid were quantified using high‐performance liquid chromatography (HPLC). These seven organic acids were all included in the VFA term for the purposes of this study. Samples taken directly from the reactor liquid were centrifuged (15 min at 11,500 g) and 700 μL of supernatant was collected. 70 μL of 5 M H_2_SO_4_ was added and samples were frozen at −20°C. After thawing, samples were centrifuged (11,500 g for 10 min), after which the supernatant was filtered using a 0.2 μm syringe filter. The filtered supernatant was analysed by HPLC on a Shimadzu 2050 Series equipped with an ion exclusion column (Rezex ROA Organic Acids H+, 300 × 7.80 mm, Phenomenex) and detected by a UV detector at a wavelength of 210 nm. The mobile phase used was 5 mM H_2_SO_4_ and the flow rate was 0.6 mL/min.

TAN was measured using Hach LCK302 Ammonium Cuvette tests and chemical oxygen demand (COD) was measured using Hach LCK514 Cuvette tests. Total solids (TS) and volatile solids (VS) of the inocula were measured according to international standard methods (A.P.H.A. 1998).

### 2.3 Growth Medium

The defined medium used in the reactor experiments was a basal medium prepared according to Westerholm et al. (2010), with additional substrates added based on Speda et al. (2016), and with varying TAN levels (Table 2). TAN levels were 1, 2 and 7 g/L for the reference SL, MN and FW reactors, and 4 g/L for all the experimental reactors, and were achieved by addition of NH_4_Cl before autoclaving. Most substrates were added to the autoclaved basal medium from stock solutions. Glucose, sucrose, cellobiose and tryptone were added directly to the medium since their solubilities did not allow for making of stock solutions.

**TABLE 2:** Substrates and their final concentrations in the defined growth medium used as feed for the bioreactors.

Substrate	Final concentration (mM)
Acetic acid	11
Propionic acid	17
Butyric acid	0.7
Formic acid	1.4
Methanol	20
Ethanol	13
Glucose	62
Sucrose	26
Cellobiose	31
Acid‐hydrolyzed casein	8
Tryptone	8

The medium was concocted to include substrates for all the biochemical steps of anaerobic digestion (hydrolysis, acidogenesis, acetogenesis and methanogenesis). The recipe was based on Speda et al. 2016, but replacing half of the amount of carbon provided by glucose with cellobiose, and half the amount of acid‐hydrolyzed casein by tryptone. The reactors were also supplemented with oleic acid by direct injection. The additions were done when the previous injection had visibly disappeared, as long‐chain fatty acids are known to cause inhibition to methanogenic communities (Kougias et al. 2016). In total, approx. 0.40 mL was added as an average weekly dose. SL and MN reactors were fed with oleic acid every 15–20 days for a total of 6 oleic acid injections each. FW reactors were fed only 3 times in the early phase of the study (the first 6 weeks) but were later not fed with more oleic acid due to incomplete degradation.

For the purpose of troubleshooting low reactor pH, after week 8 an altered version of the medium was used which contained only half of solution A (Westerholm et al. 2010) and replaced the N_2_/CO_2_ gas phase with only N_2_. This led to an increase in pH in the medium to almost 8.0, from the previous approximate 7.2.

### 2.4 Calculations and Statistical Analysis

Calculation of the maximum theoretical gas production was done using the Buswell formula (Buswell and Mueller 1952). For any calculations concerning tryptone and acid‐hydrolyzed casein, the molecular weight 133.13 g/mol was used, as the calculated average of the amino acid composition of casein (Labatut et al. 2022). The free ammonia (NH_3_) fraction of TAN was calculated according to the equation presented by Jiang et al. (2019).

Reactor pairs (SL, MN and FW) and reactor groups (reference and experimental) were compared on a multivariate basis using permutational multivariate analysis of variance (PERMANOVA). The operational parameters used for this comparison were: pH, ml of gas produced per ml substrate fed, % methane in the gas, acetate concentration and sum of other VFAs. PERMANOVA was conducted in R 4.4.2 (R Core Team 2024) using vegan 2.6.6.1 (Oksanen et al. 2024) and pairwiseAdonis 0.4.1 (Martinez Arbizu 2020). The distance matrix needed was calculated with the vegdist function using the Euclidean method. Homogeneity of multivariate dispersion was checked using the betadisper function. In all the comparisons made, multivariate dispersion was not significantly different (*p* > 0.05) between the compared groups except in one case (MN‐exp vs. MN‐ref). PERMANOVA was conducted using the adonis2 function with 10^4^ permutations. Further pairwise multilevel analysis in the case of a significant *p* value was done using the pairwise.adonis function, with 10^4^ permutations and using the Holm method (Holm 1979) for adjustment of *p* values due to multiple comparisons.

Normalised Stochasticity Ratio (NST) was calculated in R using the NST package 3.1.10 (Ning et al. 2019). For NST calculations, each reactor pair was treated as a separate metacommunity. Both taxonomic and phylogenetic NST values were calculated using the functions tNST and pNST (using default parameter values), and comparisons and descriptive statistics were calculated using the nst.boot function with 1000 random draws.

### 2.5 Microbial Community Profiling

DNA was extracted weekly from reactor samples (single replicates) with the FastDNA SPIN kit for Soil by MP Biochemicals as described by Danielsson et al. 2017. Triplicate DNA samples were also extracted from the inoculum material used for reactor start‐up. The extracted DNA samples were sent to Novogene (Cambridge, UK), which conducted library preparation and sequencing of the V4 region of the 16S rRNA gene using the Illumina Novaseq platform. The resulting sequences (primers and barcodes already removed) were analysed with the DADA2 pipeline (Callahan et al. 2016), via dada2 1.16.0 in R. Forward and reverse sequences were truncated at positions 180 and 220, based on the quality profiles generated by the pipeline. Sequencing reads that did not meet the filter criteria (maxN = 0, maxEE = c(2, 2), truncQ = 2) were not included in the following steps. Taxonomy was assigned using the Silva database 138.1 (Quast et al. 2012). Using the neighbour‐joining method, a phylogenetic tree of all the sample sequences was constructed using phangorn 2.11.1 (Schliep et al. 2017). The tree was used to generate a Principal Coordinate Analysis (PCoA) plot to study the dissimilarity of the reactor samples through the weeks of their operation. The weighted UniFrac method was used for calculating sample distances for the PCoA plot. Alpha diversity of the samples was estimated with the Shannon index, calculated in R using the plot_richness function in phyloseq 1.41.1 (McMurdie and Holmes 2013). Abundance analysis, visualisation, and graphic illustration of the results was done in R using phyloseq and ggplot2 3.5.1 (Wickham 2016). Results from a few of the DNA samples were removed from the analysis of the microbial community because they were presenting aberrant microbial composition compared to their vicinal samples. The removed samples were FW‐exp week 1, MN‐ref weeks 8 and 11, MN‐exp weeks 9 and 11, SL‐ref week 11 and SL‐exp week 11.

In addition to 16S rDNA analysis, taxonomic profiling based on complete metagenomic reads was performed on a subset of the samples. Metagenomic reads were processed using fastp 0.20.0 to remove poor quality bases (<Q20), and adapters were removed using the same software (Chen 2023). After these preprocessing steps, 22–150 M raw reads were kept per sample. For taxonomic profiling, remaining reads were classified using Kraken2 2.1.2 (Wood et al. 2019) using the GTDB r95 database (Parks et al. 2022) prepared by De La Cuesta‐Zuluaga et al. (2020). Species abundance was calculated using Bracken 2.6.2 (Lu et al. 2017). A phylogenetic tree was constructed using the bac120 taxonomy and tree files provided by GTDB using gracken, a tool which was custom‐developed for this purpose (Ohlsson 2025). Briefly, the GTDB tree was loaded using ETE 3.1.3 (Huerta‐Cepas et al. 2016) and pruned to only contain the type genomes for the species present in the Bracken report files. A similar tree was constructed for the archaeal (ar122) species, and the two trees were then combined. The combined tree was loaded into R using ape 5.8 (Paradis and Schliep 2019). PCoA plots of the microbial communities were generated using phyloseq 1.50.0 using the weighted UniFrac distance metric. Krona plots (Ondov et al. 2011) of species abundance were generated using KrakenTools (commit hash d4a2fbe) (Lu et al. 2022) after filtering out species below 0.01% abundance. Sankey diagrams were generated using Pavian 1.2.1 (Breitwieser and Salzberg 2020).

For functional analysis, SUPER‐FOCUS 1.4.1 (Silva et al. 2016) was used for mapping metagenomic reads to their corresponding SEED subsystems. The DB_100 UniRef database was used, with DIAMOND 2.0.15 (Buchfink et al. 2021) as the aligner, and using the default normalisation, which distributes reads equally over matching subsystems in the case of multiple matches. Results were visualised using ggplot2 and pheatmap 1.0.13 (Kolde 2025), using the *complete linkage* method for clustering.

## 3 Results

### 3.1 Reactor Operation

Six different reactors were inoculated with 3 different inocula (Table 1) and operated for a total of 12 weeks (3 HRT). TAN levels at the end of the operation of all reactors were close to their intended values. Chemical parameters for all reactors during the final 5 weeks of operation are presented in Table 3.

**TABLE 3:** Operational data for the 6 reactors included in the study during their last 5 weeks of their operation, presented as the mean value and its standard deviation.

	SL		MN		FW	
	Reference	Experimental	Reference	Experimental	Reference	Experimental
pH	6.97 ± 0.06	6.87 ± 0.02	7.01 ± 0.06	6.90 ± 0.13	6.71 ± 0.24	7.11 ± 0.16
Total VFA (g/L)	0.06 ± 0.04	0.51 ± 0.36	0.17 ± 0.15	0.32 ± 0.22	3.66 ± 1.13	2.12 ± 0.64
Acetate (g/L)	0.03 ± 0.01	0.26 ± 0.19	0.07 ± 0.10	0.24 ± 0.19	2.36 ± 0.99	1.19 ± 0.48
TAN (g/L)	1.02 ± 0.04	3.85 ± 0.13	1.97 ± 0.07	3.71 ± 0.09	7.13 ± 0.52	4.63 ± 0.27
Gas production (mL/mL added substrate)	25.01 ± 1.50	23.79 ± 3.05	22.54 ± 2.73	21.83 ± 1.29	22.91 ± 3.93	26.78 ± 2.76
CH_4_ in biogas (%)	49.70 ± 2.26	47.83 ± 3.07	46.08 ± 3.27	46.39 ± 1.26	40.72 ± 9.02	48.71 ± 3.33
% of theoretical gas yield	85.3 ± 5.10	81.1 ± 10.4	76.9 ± 9.30	74.4 ± 4.40	78.1 ± 13.4	91.3 ± 9.40

*Note:* Gas production was measured weekly, and periods of no feeding are excluded from the measurement. Oleic acid is not included in the calculation of the theoretical amount of gas, based on the Buswell formula. Total VFA includes acetic, propionic, butyric, isobutyric, valeric, isovaleric and lactic acid.

All reactors showed fairly stable gas production over the operation period, reaching 74%–92% of the theoretical amounts (Table 3). The SL and MN reactors showed some VFA accumulation after 60 days of operation, but concentration never exceeded 1.2 g/L, and the levels decreased towards the end of the 12 weeks of operation (Figure S1). Although pH for these four reactors was slowly decreasing over the course of the experiment, this was not associated with VFA accumulation (Figure S1). FW‐exp experienced an acidification event (days 28–35) with a sudden decrease in pH and a concomitant increase in VFAs (mainly acetate), consequently resulting in inhibition of methanogenesis. To allow for VFA degradation, feeding was stopped for 6 days. This allowed the process to recover its gas production, and no further accumulation of VFAs was observed (Figure S1). Overall, the FW reactors exhibited more acidification and lower pH levels compared to the other reactors and showed higher VFA levels.

Multivariate comparison of the reactors using PERMANOVA was done to evaluate differences in the reactors' operational performance, using the operational values of the last 5 weeks of the study (Table 3, Tables S1–S2). There were no statistically significant differences in the performance of the reference reactors (*p* = 0.084), while the experimental reactors differed significantly (*p* = 0.046). *Post hoc* testing revealed significant differences only between FW and MN in the experimental group (Table S1), with the difference mostly being higher gas production and VFA accumulation in FW‐exp. Significant differences in the operation of each reactor pair were also investigated on a multivariate basis using PERMANOVA, finding no statistically significant changes within the pairs (Table S2).

### 3.2 Microbial Community Profile

**FIGURE 1:**
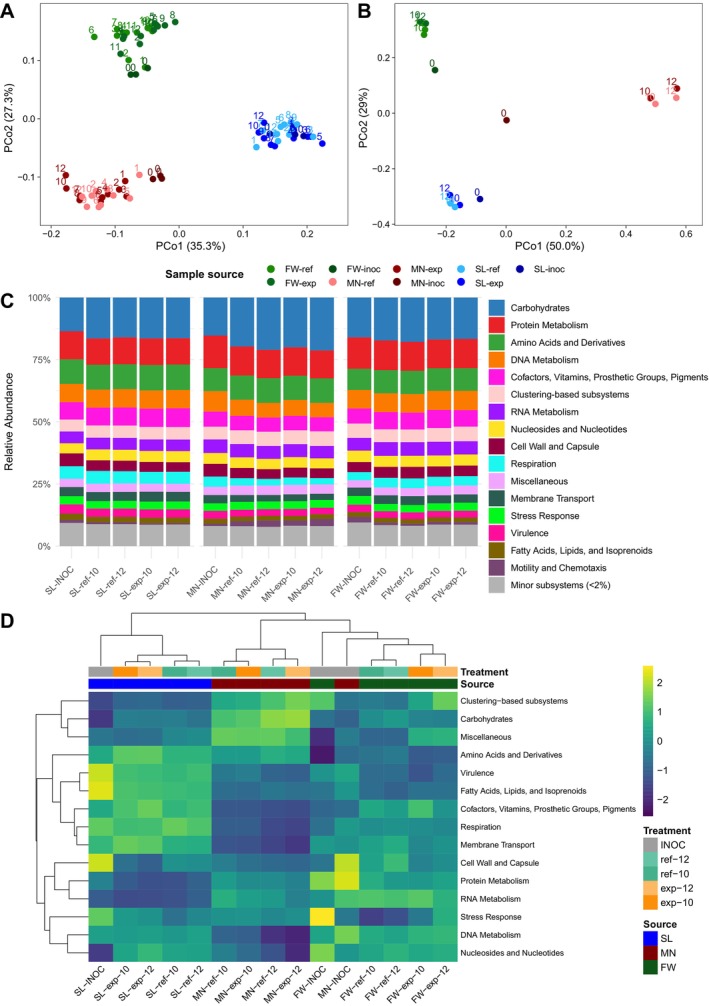
Taxonomic and functional composition of reactor samples and inocula. PCoA plots of phylogenetic distances between all the reactor samples and the samples from the inoculum material, based on the Weighted UniFrac method on the 16S (A) or metagenomic (B) sequencing data. Numbers top left of each point indicate weeks since inoculation. Inoculum samples are labelled as 0. (C) Functional profiles of reactor communities and inocula classified at SEED subsystem level 1 visualised using relative abundances. (D) Row‐scaled heatmap visualising the functional composition of the reactor samples using the 15 most abundant SEED subsystem level 1 categories. Samples are either reference (ref), experimental (exp), or corresponding inocula, optionally suffixed with weeks since inoculation.

The microbial communities of the inocula and the weekly reactor samples were taxonomically profiled by 16S rRNA and metagenomic sequencing, and the metagenomic sequences were functionally profiled on multiple levels of functional resolution (Figures 1 and S4–S10). Taxonomically, reactor communities were clearly separated based on their respective inocula, but irrespective of TAN concentrations. According to the 16S taxonomic profile, SL reactor communities clustered closely to their inocula, compared with the FW and MN reactor communities, which presented somewhat more dispersed clusters, showcasing some variation throughout their operation (Figure 1A). In the taxonomic metagenome analyses, SL and FW reactor samples clustered near their respective inocula, with FW‐exp and FW‐ref diverging a bit more from their inoculum community than SL‐exp and SL‐ref from SL inoculum, while MN‐ref and MN‐exp diverged significantly from MN inoculum, while remaining fairly similar to one another.

Despite taxonomic differences, only small functional differences could be seen at the broadest functional classification level (SEED subsystem level 1; Figure 1C). Differences in functional profiles of the reactor communities largely reflected changes in taxonomy, with SL communities diverging the least, and MN the most, from their inocula (Figures 1D and S8–S10). Clustering was similar between taxonomic and functional results, with PCA plots of the functional composition (Figure S10) largely mirroring taxonomic PCoA plots (Figure 1A,B). Taken together, the microbial communities of all experimental reactors were decidedly more similar to those of their respective reference reactors than to those of the experimental reactors of the other inocula, despite similar growth conditions, feeding substrate, and TAN concentrations. Variation in community composition between reference and experimental reactors within each pair was minimal. Final microbial community composition appeared to be more strongly influenced by the inoculum source than by the applied treatment conditions, including substrate type and TAN concentration.

### 3.3 Microbial Community Development

The three microbial community pairs in our study exhibited varying degrees of divergence from their original composition. The following section provides a summary of the most prominent taxonomic changes observed between the inocula and their descendant communities.

**FIGURE 2:**
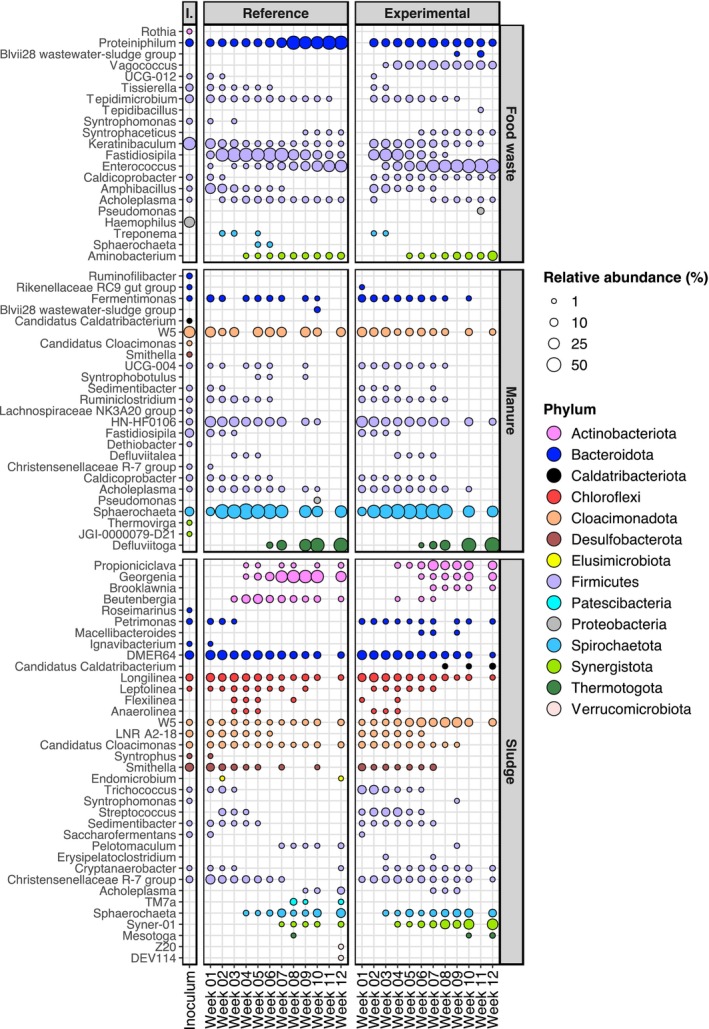
Relative abundance of different bacterial genera in reactor samples over the course of their operation, coloured based on their respective phyla. The size of each point is representative of the relative abundance of this genus in the sample. Each panel represents a reactor, labelled based on its inoculum (FW, MN and SL), and the TAN concentration they were subjected to (reference or experimental). There is one replicate of each reactor sample. The composition of the inocula is presented in a column to the left, where triplicate samples were averaged out and presented as one. On the x axis, each sample is represented by the number of weeks from the start of their operation (1–12). The genera shown have more than 1% relative abundance. Genera with lower than 1% relative abundance are not shown.

**FIGURE 3:**
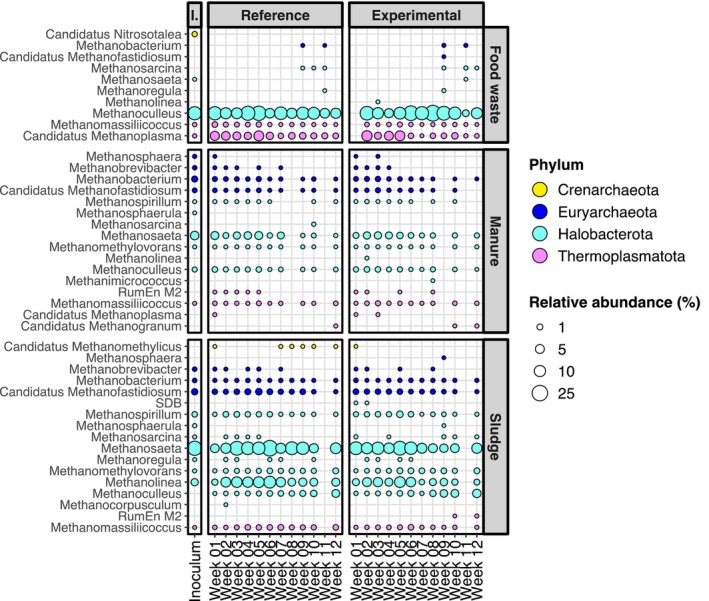
Relative abundance of different archaeal genera in reactor samples over the course of their operation, coloured based on their respective phyla. The size of each point is representative of the relative abundance of this genus in the sample. Each panel represents a reactor, labelled based on its inoculum (FW, MN and SL), and the TAN concentration they were subjected to (reference or experimental). There is one replicate of each reactor sample. The composition of the inocula is presented in a column to the left, where triplicate samples were averaged out and presented as one. On the x axis, each sample is represented by the number of weeks from the start of their operation (1–12).

The SL reactors differentiated minimally from their inoculum community. Along with SL inoculum, they exhibited a relatively equal abundance of several different bacterial phyla (Figures 2, S2 and S4), such as Firmicutes, Cloacimonadota, Bacteroidota, Halobacterota and Chloroflexi. Over the course of their operation, both SL reactors experienced an increase in relative abundance in genera of the phylum Actinobacteriota and in the genus Syner‐01 (family Synergistaceae) and a decrease in the genus Smithella (Figure 2). The methanogenic population in the SL reactors was dominated, according to 16S analysis, by the genera Methanosaeta and Methanolinea (Figure 3). During the final weeks of the study, the genus Methanoculleus increased in abundance in both SL reactors (although with relative abundance < 4%). The metagenomic analysis proposed a more marked increase of the genus Methanoculleus, from 0.4% in the inoculum to 10% in SL‐ref and 12% in SL‐exp (Figures S4 and S5; File S2).

The minor dissimilarities seen in the composition between FW reactors and inoculum, according to the 16S analysis, could be attributed to various genera in the phylum Firmicutes, the genera Aminobacterium and Proteiniphilum, and the phylum Cloacimonadota (Figures 2 and S2). However, larger differences were observed in the taxonomic analysis of the metagenome, reflected in the somewhat higher degree of divergence seen in the metagenome PCoA (Figure 1B). According to this analysis, genera Aminobacterium and Tepidanaerobacter exhibited a marked increase in both FW‐exp and FW‐ref compared to the inoculum (Figures S4 and S7; File S2). The methanogenic community of the FW reactors remained stable over the course of the study and was comprised mainly of the genus Methanoculleus and the genus *Candidatus* Methanoplasma (family Methanomethylophilaceae; Figure 3).

The majority of the bacterial abundance of the MN inoculum was represented by various members of different phyla, namely Bacteroidota, Cloacimonadota, Firmicutes and Spirochaerotota (Figures 2, S4 and S6; File S2). Based on 16S analysis, MN reactors demonstrated minor taxonomic divergence from their inoculum, with a dominance of genus Spirochaeta. However, a large increase in relative abundance of the genus Defluviitoga was observed during the final 4 weeks, rising to 17%–60% of sequence reads (below 1% initially) (Figure 2). The metagenomic analysis showed a greater divergence between MN inoculum and reactors, but in line with the 16S analysis, *Defluviitoga tunisiensis* (phylum Thermotogota) was the most dominant species of the MN reactors, representing approximately 60%–80% of all metagenomic sequence reads. Regarding the methanogenic community, the 16S analysis demonstrated an even and stable distribution of abundances between genera of three phyla: Euryarchaeota, Halobacterota and Thermoplasmatota (Figure 3). The metagenome analysis attributed approximately half of the archaeal relative abundance to the genus Methanoculleus and revealed a substantial increase in genera of the family Methanomassiliicoccaceae in both MN reactors compared to their inoculum (Figures S4 and S6; File S2).

### 3.4 Microbial Community Assembly Processes

**FIGURE 4:**
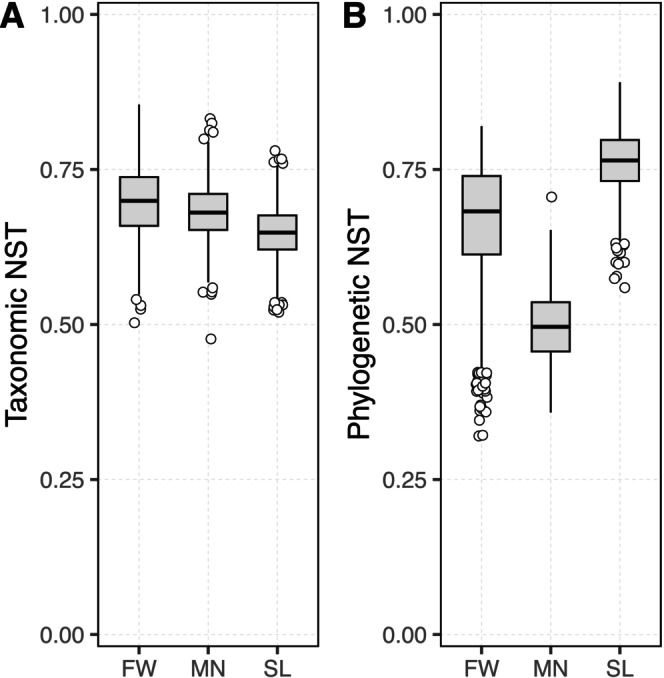
Descriptive statistics of Normalised stochasticity ratio (NST) values of the study's three metacommunities SL, FW and MN, derived from bootstrapping. Community similarity was assessed using taxonomic dissimilarity (A) and phylogenetic beta diversity (B). Boxplots show the median (line) and 1st/3rd quartiles (hinges), with whiskers extending to non‐outlier values. Circles signify outlier values (> 1.5 * IQR).

The assembly processes that possibly influenced the development of the microbial communities were characterised using the normalised stochasticity ratio (Figure 4). When the similarity of the reactor communities was assessed based on taxonomy (Figure 4a), the metacommunities exhibited NST values equal to 70% (FW), 65% (SL) and 68% (MN), all greater than 50%. This indicated that stochastic processes played a more significant role in the assembly of the reactor communities than deterministic processes. When phylogeny was also taken into account for assessing community similarity, the metacommunities exhibited NST values equal to 70% (FW), 79% (SL) and 50% (MN). In the case of FW and SL, once again it was indicated that community structure was shaped predominantly by stochastic rather than deterministic forces. The NST value for the MN metacommunity in this comparison demonstrated that determinism and stochasticity possibly participated equally in the assembly process.

## 4 Discussion

### 4.1 Taxonomic Distribution of the Microbial Communities in the Different Inocula

The taxonomic compositions of the inocula chosen for the present study were distinctly different, as was previously reported for AD communities operating with different substrates and ammonia concentrations (Calusinska et al. 2018; De Vrieze, Saunders, et al. 2015). The FW inoculum had significantly lower microbial diversity, as indicated by its Shannon index (Figure S3), while having the highest TAN concentration at 7.17 g/L. A negative correlation between microbial diversity and ammonium/free ammonia concentrations has been reported previously (Calusinska et al. 2018; Li et al. 2015). The distribution of microbial phyla observed in the inocula was also consistent with earlier studies. Firmicutes and Bacteroidota are often dominant phyla in manure‐based bacterial communities (Li et al. 2015), with Firmicutes being more prevalent in manure‐based than in sludge‐based processes. Chloroflexi has also been previously found in mesophilic sludge communities (Sundberg et al. 2013). In reactors with high‐TAN concentrations operating on food waste, Firmicutes has been shown to dominate the microbial population, with up to 80% relative abundance (Müller et al. 2016).

The distribution of methanogenic genera in the inocula was consistent with their respective TAN concentrations (Figures 3 and S4). The SL and MN inocula contained a range of hydrogenotrophic, methylotrophic and acetoclastic methanogenic genera, whereas the high‐TAN FW inoculum was dominated by the hydrogenotrophic genus Methanoculleus, a key methanogen in high‐ammonia AD systems (reviewed in Capson‐Tojo et al. 2020). Candidatus Methanoplasma, a member of the methylotrophic Methanomassiliicoccales order (Adam et al. 2017), was also present in the FW inoculum, consistent with other studies that have found this genus enriched in high‐ammonia reactors (Lendormi et al. 2022).

### 4.2 Substrate Did Not Deterministically Influence Final Community Structure

Some previous studies have reported that microbial communities in AD converge taxonomically and functionally when provided with the same substrate (De Vrieze, Gildemyn, et al. 2015; Duan et al. 2021; Liu et al. 2017; Peces et al. 2018), and others have found taxonomically similar inocula to diverge when given different substrates (De Francisci et al. 2015; Eliasson et al. 2023). These findings have supported the view that substrate composition is a key deterministic factor in shaping community structure.

In contrast, we found no evidence for taxonomic convergence when different inocula were provided with the same defined medium. Null‐model analysis (NST) indicated that stochastic processes dominated community assembly, and we found final community composition to be primarily determined by inoculum composition. This aligns with Han et al. 2016, who also observed a lack of convergence in reactors fed with a defined medium, and, highlighting stochastic processes, found only ~30% similarity between replicate reactors.

One explanation for the discrepancy with earlier studies may lie in the absence of strong selective pressures in our system. Convergence is often reported under conditions with potent drivers of community composition, such as thermophilic temperature or high free ammonia, which select for highly specialised organisms (De Vrieze, Saunders, et al. 2015; Duan et al. 2021). Our medium contained a variety of substrates and no dominant inhibitory or selective agent, with free ammonia levels similarly low across reactors, leaving a broad metabolic niche for taxonomic changes to occur within. In our study, stochastic events most likely led to the prevailing of some microorganisms over others. For example, *D. tunisiensis* was detected through metagenomic analysis in all the inoculum communities (relative abundance 0.03%–0.2%, Figure S5), but was enriched only in the MN reactors. Thus, the overrepresentation of *D. tunisiensis* in MN reactors could not be solely explained by a possible deterministic influence of the chemical characteristics of the medium but might rather have been caused by stochastic events.

### 4.3 Ecological Pressures Conserve Functionality Rather Than Taxonomy

Comparing the functional composition of the communities, certain metabolic functions were universally reduced in abundance compared to the inocula over the course of the experiment (e.g., sulfur metabolism, protein metabolism), reflecting changes to the metabolic environment compared to native conditions. At higher classification resolution, more specific shifts were observed; for example, the SL inoculum was enriched in metabolic traits specific to its native milieu (e.g., anaerobic degradation of aromatic compounds, hydrocarbon metabolism; Figure S9), traits that were considerably less abundant at the end of the experiment.

Despite only small differences in functional profiles, taxonomic differences between reactor pairs were pronounced. This is consistent with findings in other ecosystems, such as the human microbiome (Huttenhower et al. 2012; Turnbaugh et al. 2009) and bromeliad central cavities (Louca et al. 2016). Given the comparable reactor performances, this suggests that selection pressures acted more strongly on maintaining functional potential than on conserving specific taxa in the thermodynamically constrained environment. This also helps explain the strong dominance of *D. tunisiensis* in the MN reactors, despite the absence of major functional or operational shifts. Owing to its versatile carbohydrate metabolism and its acetogenic and hydrogenogenic capabilities (Ben Hania et al. 2012; Maus et al. 2016), this species is capable of carrying out nearly all steps of anaerobic digestion, except methanogenesis, on many of the supplied substrates.

Although the communities were taxonomically distinct yet broadly similar in function, principal component analysis of their functional profiles produced ordinations closely resembling the taxonomic PCoA plots, indicating a strong link between taxonomy and function at finer classification levels. This apparent paradox might stem from high‐resolution functional classifications effectively acting as genomic fingerprints. In the MN samples, for example, the high relative abundance of *D. tunisiensis* exerts a disproportionate influence on apparent community‐level functionality through its versatile carbohydrate metabolism (e.g., bottom left corner of Figure S9). This may partly explain why functional annotation often is no better than taxonomic data for classification accuracy of microbial communities (Boers et al. 2025; Xu et al. 2014).

### 4.4 TAN Concentration Had Limited Impact on the Progression of Microbial Communities

In contrast to many other studies (reviewed in Capson‐Tojo et al. 2020), the concentration of TAN had limited impact on the separation of each reactor pair (Figure 1). Inhibition by TAN has been well characterised and shown to primarily impact the composition of the methanogenic community (Hardy et al. 2021; Schnürer and Nordberg 2008). It has been suggested that the shift from acetoclastic to hydrogenotrophic methanogenesis, known to occur under ammonia inhibition conditions, is primarily driven by free ammonia and not TAN concentration (Capson‐Tojo et al. 2020; Jiang et al. 2019; Schnürer and Nordberg 2008). Conversely, there is also evidence for NH_4_^+^ concentration making the major contributions to inhibition instead of free ammonia, in environments with pH lower than 7 (Astals et al. 2018). In the present study, all reactors operated at neutral pH (6.8–7), which kept free ammonia concentrations well below 0.1 g/L, a concentration mostly accepted as non‐inhibitory (Capson‐Tojo et al. 2020). This level of free ammonia concentration is unlikely to have driven the changes observed in the methanogenic communities of the SL and MN reactors. Additionally, TAN concentration (and therefore also NH_4_^+^ concentration) could also be ruled out as the major determinant for the changes in the methanogenic communities, as the changes happened symmetrically in the reference reactors compared to their respective experimental reactors. Regardless, the SL reactors showed an increase in the relative abundance of the hydrogenotrophic methanogen Methanoculleus, despite free ammonia concentration well below 0.1 g/L, and with no apparent operational inhibition. Other factors, e.g., substrate composition, degree of conversion, and/or low pH (relative to common AD processes), might have been more important for the increase of this hydrogenotrophic methanogen. Acetate has been suggested to account for approx. 70% of methane produced in sludge‐based biogas processes, which are consequently typically enriched with acetoclastic methanogens, specifically Methanosaeta (Jiang et al. 2018). The decrease of Methanosaeta and concomitant increase of Methanoculleus in SL reactors suggests either a decreased production of acetate as compared to the sludge process from which the inoculum was taken, or, alternatively, SAOB outcompeting acetoclastic methanogens. The relative abundance of bacterial genus Syner‐01 (family Synergistaceae) increased in both SL reactors. Members within this family and genus have been proposed to perform syntrophic acetate oxidation in sludge‐based processes and to be more competitive for acetate over Methanosaeta at high acetate concentrations (2.5–10 mM) (Ito et al. 2011; Zhang et al. 2022). Increasing acetate concentrations combined with low pH (Figure S1) could have contributed to a diversification of acetate‐degrading pathways in these reactors.

The FW reactors started with elevated concentrations of free ammonia (0.85 g/L) and slowly transitioned to levels below 0.1 g/L. Interestingly, this did not induce a “reversal” of the shift from acetoclastic to hydrogenotrophic methanogenesis. Additionally, the decrease in TAN levels in FW‐exp also appeared to have no effect on its methanogenic community. This might be evidence that once the shift to SAO has occurred, it is not easily reversed, even if the free ammonia concentration returns to non‐inhibitory levels. The regime shift and stability of microbial communities exposed to increasing ammonia levels in biogas processes have been extensively studied; however, the resilience of ammonia‐inhibited communities to low ammonia conditions has, to our knowledge, not been studied before. However, the results are in line with studies of soil communities, revealing that microbial communities affected by stress disturbances generally do not recover their pre‐disturbance composition (Shade et al. 2012). Additionally, the acidification of the FW reactors suggested that despite the stable hydrogenotrophic methanogenic population, acetate utilisers in the community were not performing well. It is possible that the SAOB present in the FW reactors, Syntrophaceticus and Tepidanaerobacter, which have been suggested to be positively impacted by high free ammonia concentrations (Manzoor et al. 2018; Westerholm et al. 2019), were hampered in low free ammonia conditions.

## 5 Conclusions

The findings of this study propose that inoculum source, and not substrate composition, was the main contributing factor for the microbial community assembly in lab‐scale biogas reactors. In contrast with previous studies, the chemical characteristics of the feeding substrate failed to cause a taxonomic convergence in a deterministic manner in three cultures derived from different biogas inocula. Additionally, TAN concentration appeared to not influence community structure, since taxonomic shifts occurred symmetrically in reactors differing in TAN concentrations. The observation that taxonomically divergent communities remained largely functionally similar suggests that ecological pressures were exerted more strongly on function than taxonomy. Moreover, a microbial community initially adapted to high TAN, and apparently operating with syntrophic acetate oxidation‐driven methanogenesis, appeared “locked in” and did not change as expected with decreasing TAN levels. A null model investigation of the microbial assembly process of the study's microbial communities suggested that stochastic processes, rather than deterministic ones, played a more significant role in shaping their composition over time.

## Author Contributions

**Vasiliki Tsamadou:** investigation, writing – original draft, writing – review and editing, formal analysis, data curation, visualization, methodology, validation. **Jonas A. Ohlsson:** writing – review and editing, formal analysis, data curation, visualization, methodology, validation. **Anna Schnürer:** conceptualization, funding acquisition, writing – review and editing, investigation, supervision, resources, project administration, methodology.

## Conflicts of Interest

The authors declare no conflicts of interest.

## Supporting information


**Data S1:** Supplementary Figures and Tables.

**File S2:** Krona plot of microbial community composition in inocula and reactors.

## Data Availability

16S and metagenomic sequence reads were deposited at NCBI under the BioProject PRJNA1242372. The software for creating the metagenomic tree is available at https://github.com/jonasoh/gracken (doi: 10.5281/zenodo.15064484).
