# Supplementary material for: Converge or Diverge? Exploring the Fate of Taxonomically Different Anaerobic Digestion Communities Under Uniform Growth Conditions
Source: Microb Biotechnol. 2025 Sep 24;18(9):e70233. doi: 10.1111/1751-7915.70233 (PMC12457802; doi:10.1111/1751-7915.70233)
Supplement: Supplementary file 1 — Data S1: Supplementary Figures and Tables. [file MBT2-18-e70233-s002.pdf]

Supplementary Material for *Converge or diverge? Exploring the fate of taxonomically different anaerobic digestion communities under uniform growth conditions*

Vasiliki Tsamadou, Jonas A. Ohlsson, and Anna Schnürer

Author affiliation:

Department of Molecular Sciences, BioCenter, Box 7025, Swedish University of Agricultural Sciences, Uppsala, S-750 07 Sweden

Corresponding Author: Anna Schnürer, anna.schnurer@slu.se, +46734231517

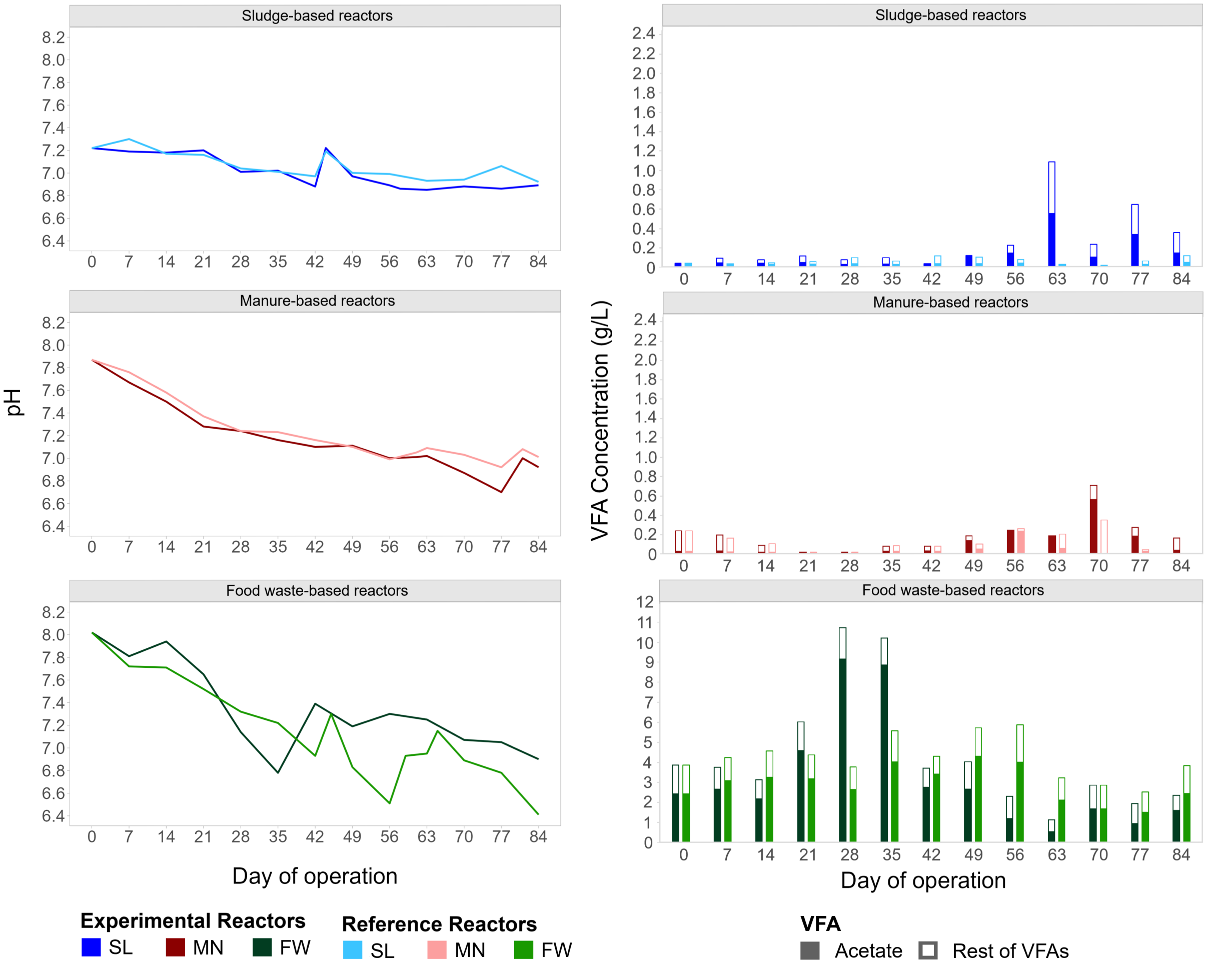

Figure S1: The progression of pH and VFA concentration over the course of the 12 weeks of reactor operation. "Rest of VFAs" includes the sum of the concentration of the following short-chain fatty acids; lactic, propionic, butyric, iso-butyric, valeric, and iso-valeric acid. Please take note that the y axis for the manure and sludge panels for VFA concentration ranges from 0 to 2.4 g/L, while for food waste the y axis ranges from 0 to 12 g/L. The reactors were initiated with inoculum from sludge (SL), manure (MN) and food waste (FW) based biogas processes. The experimental reactors were all operated at 4 g/L total ammonia nitrogen (TAN) and the reference reactors were operated at a TAN corresponding to levels in the initial inoculums, 1, 2, and 7 g/L for SL, MN and FW, respectively.

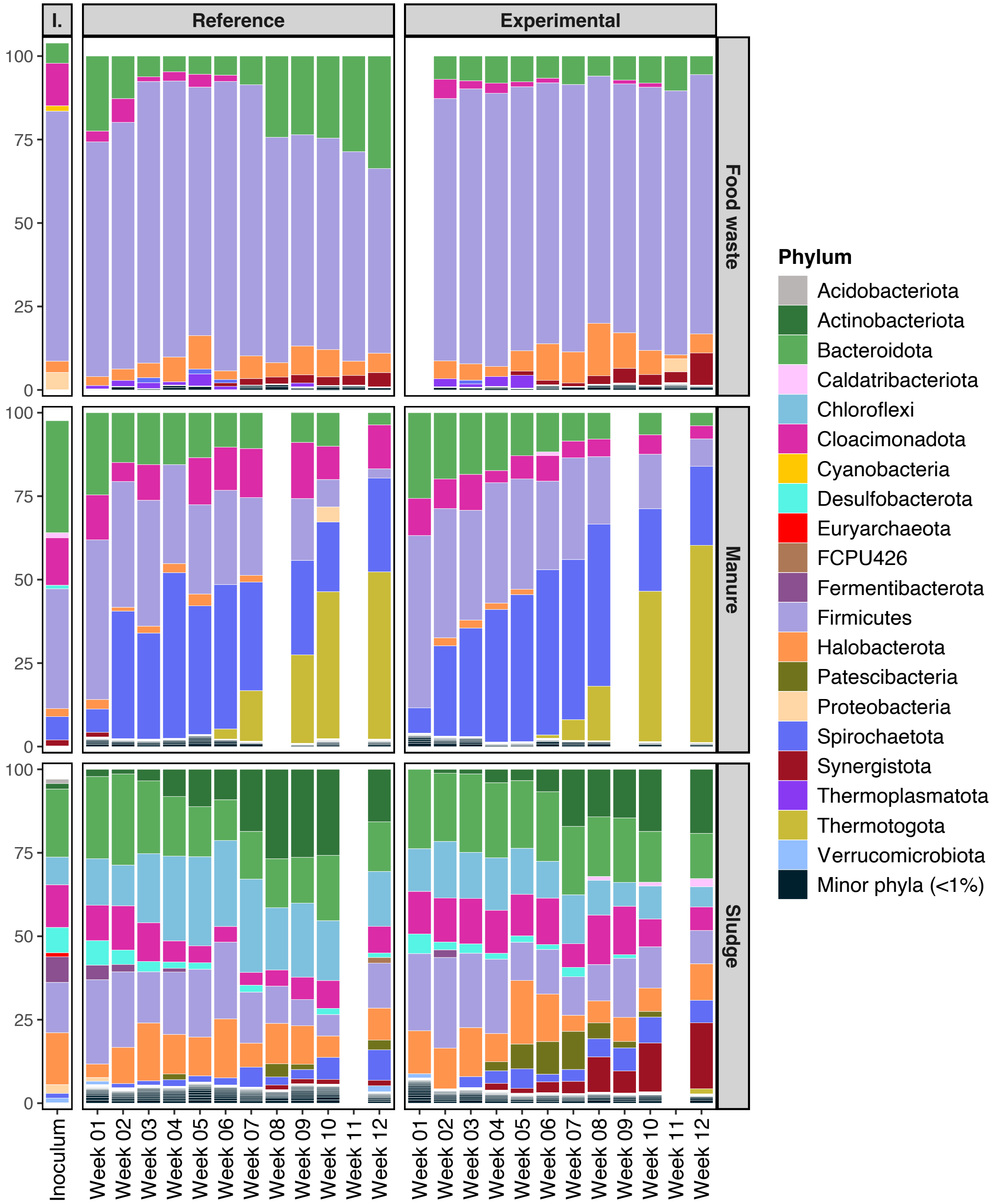

Figure S2: Relative abundance of different phyla in reactor samples over the course of their operation. Each panel represents a reactor, labelled based on inoculum (FW, MN and SL) and the TAN concentration they were subjected to (reference or experimental). There is one replicate of each reactor sample. The composition of the inocula is presented in a column to the left, where triplicate samples were averaged out and presented as one. On the x axis, each sample is represented by the number of weeks from the start of their operation (1–12). The phyla shown have more than 1% relative abundance.

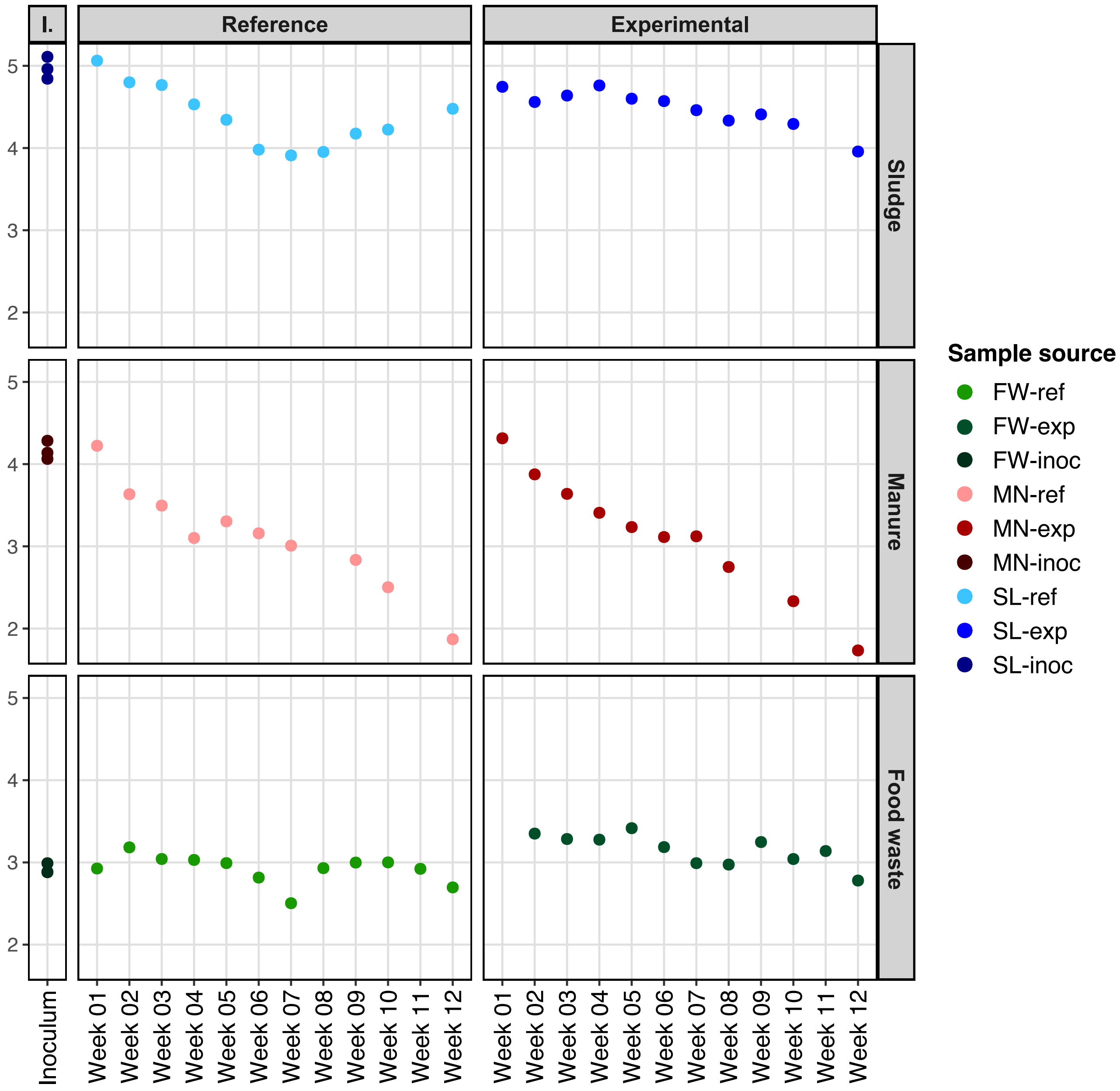

Figure S3: The Shannon diversity index calculated for reactor samples over the course of their operation. Each panel represents a reactor, labelled based on inoculum (FW, MN and SL) and the TAN concentration they were subjected to (reference or experimental). There is one replicate of each reactor sample. The index of the inocula is presented in a column to the left, where triplicate samples are shown. On the x axis, each sample is represented by the number of weeks from the start of their operation (1–12). The diversity index is not shown for aberrant samples excluded from the analysis.

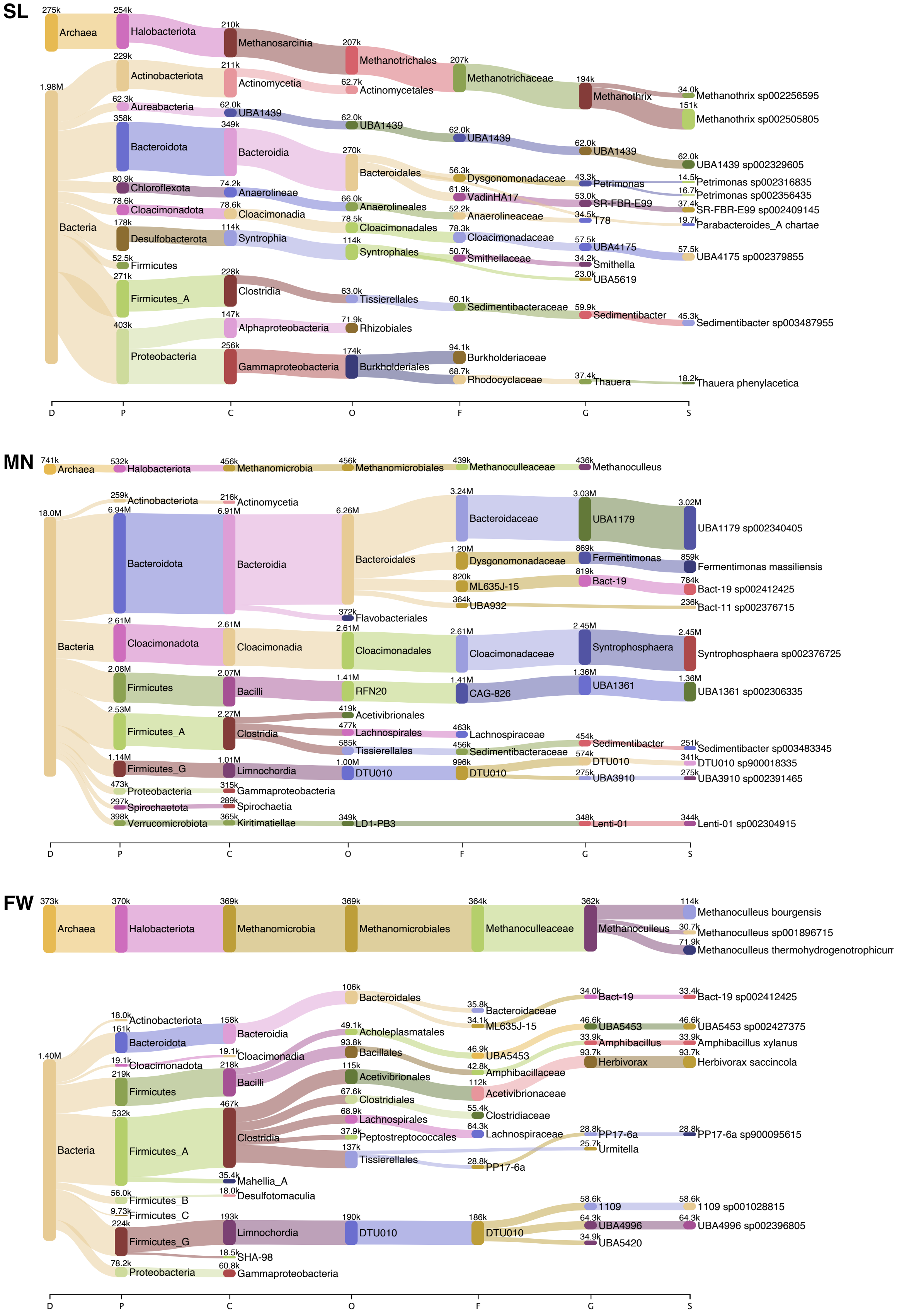

Figure S4. Sankey diagrams showing taxonomic composition of the inocula used for initiation of the bioreactors, taken from sludge (SL), manure (MN) and food waste (FW) based biogas processes.

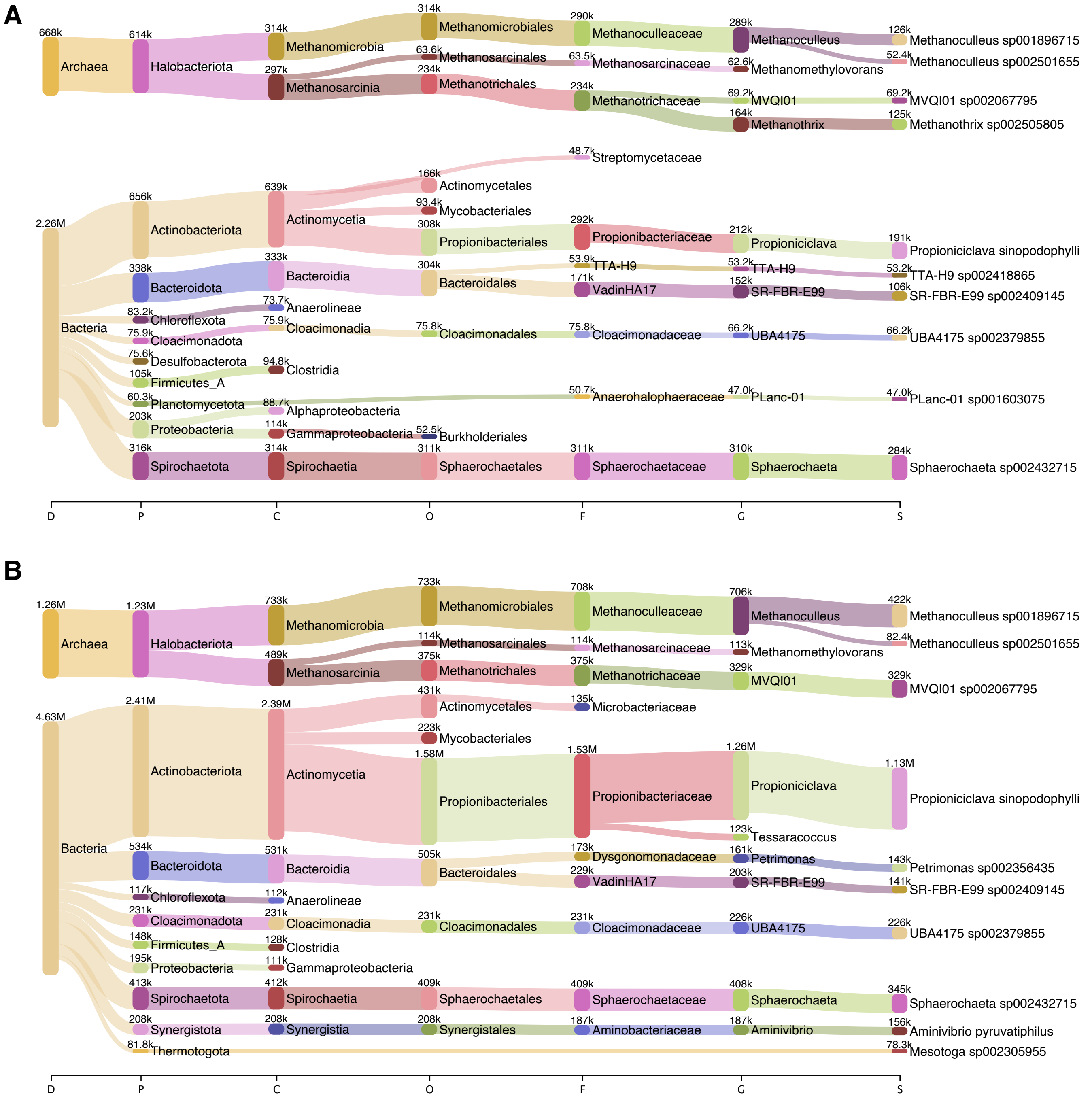

Figure S5. Sankey diagrams showing taxonomic composition in the reactors initiated with an inoculum from a sludge-based biogas process (SL) after 84 days of operation. Reference (A) and experimental (B) reactors, operated at 1 and 4 g/L total ammonia nitrogen (TAN), respectively.

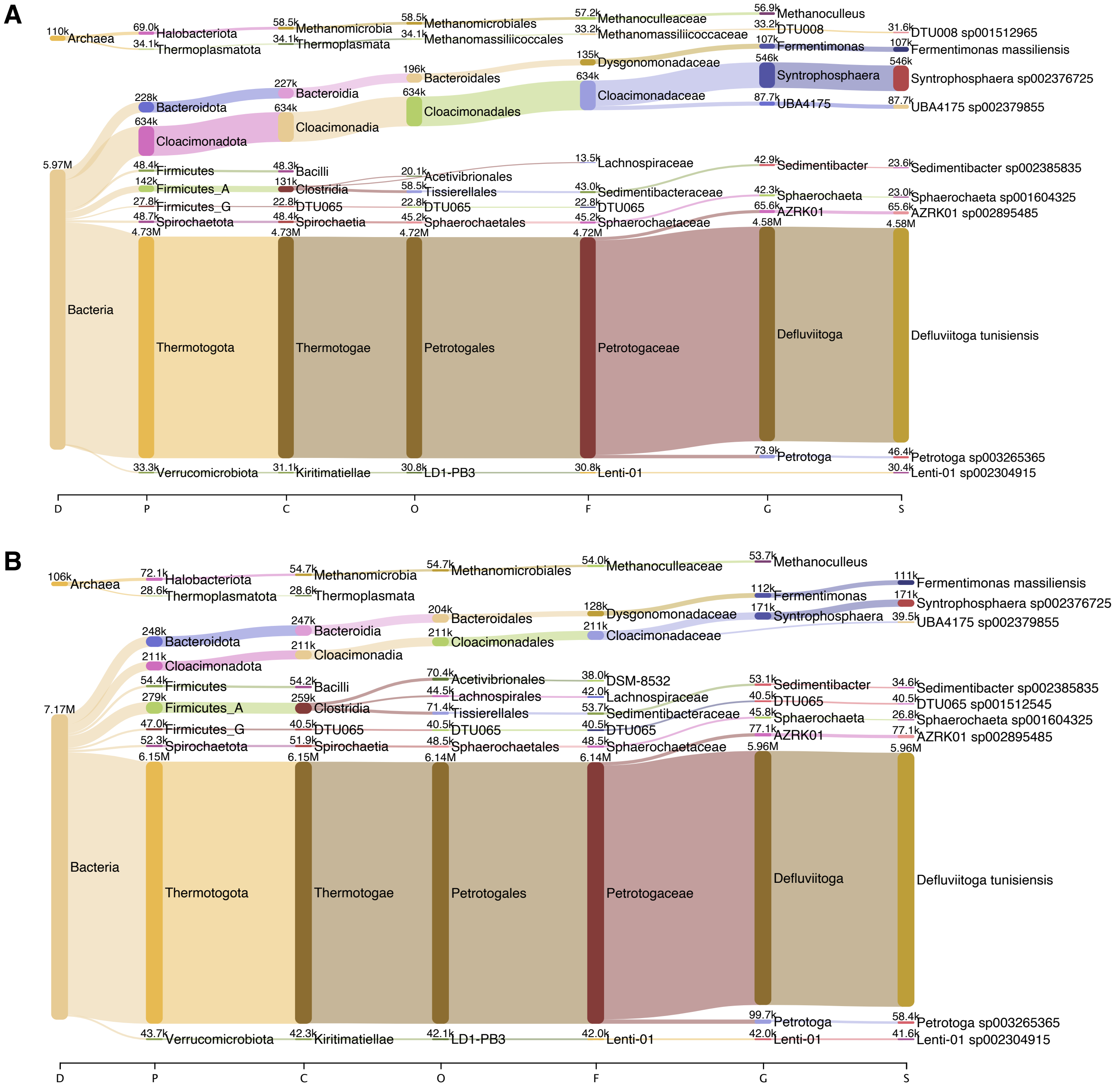

Figure S6. Sankey diagrams showing taxonomic composition in the reactors initiated with an inoculum from a manure-based biogas process (MN) after 84 days of operation. Reference (A) and experimental (B) reactors, operated at 2 and 4 g/L total ammonia nitrogen (TAN), respectively.

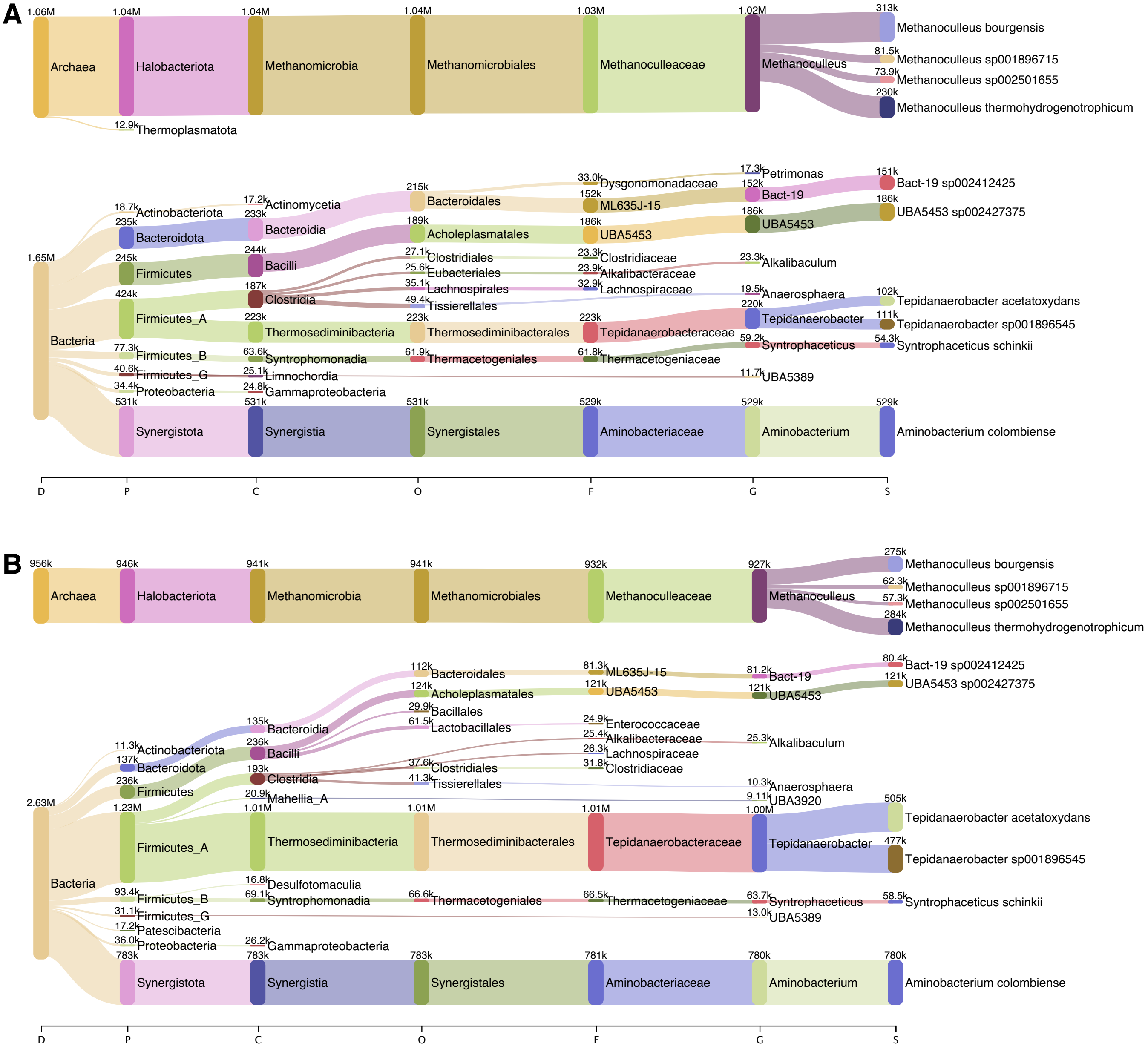

Figure S7. Sankey diagrams showing taxonomic composition in the reactors initiated with an inoculum from a food waste-based biogas process (FW) after 84 days of operation. Reference (A) and experimental (B) reactors, operated at 7 and 4 g/L total ammonia nitrogen (TAN), respectively.

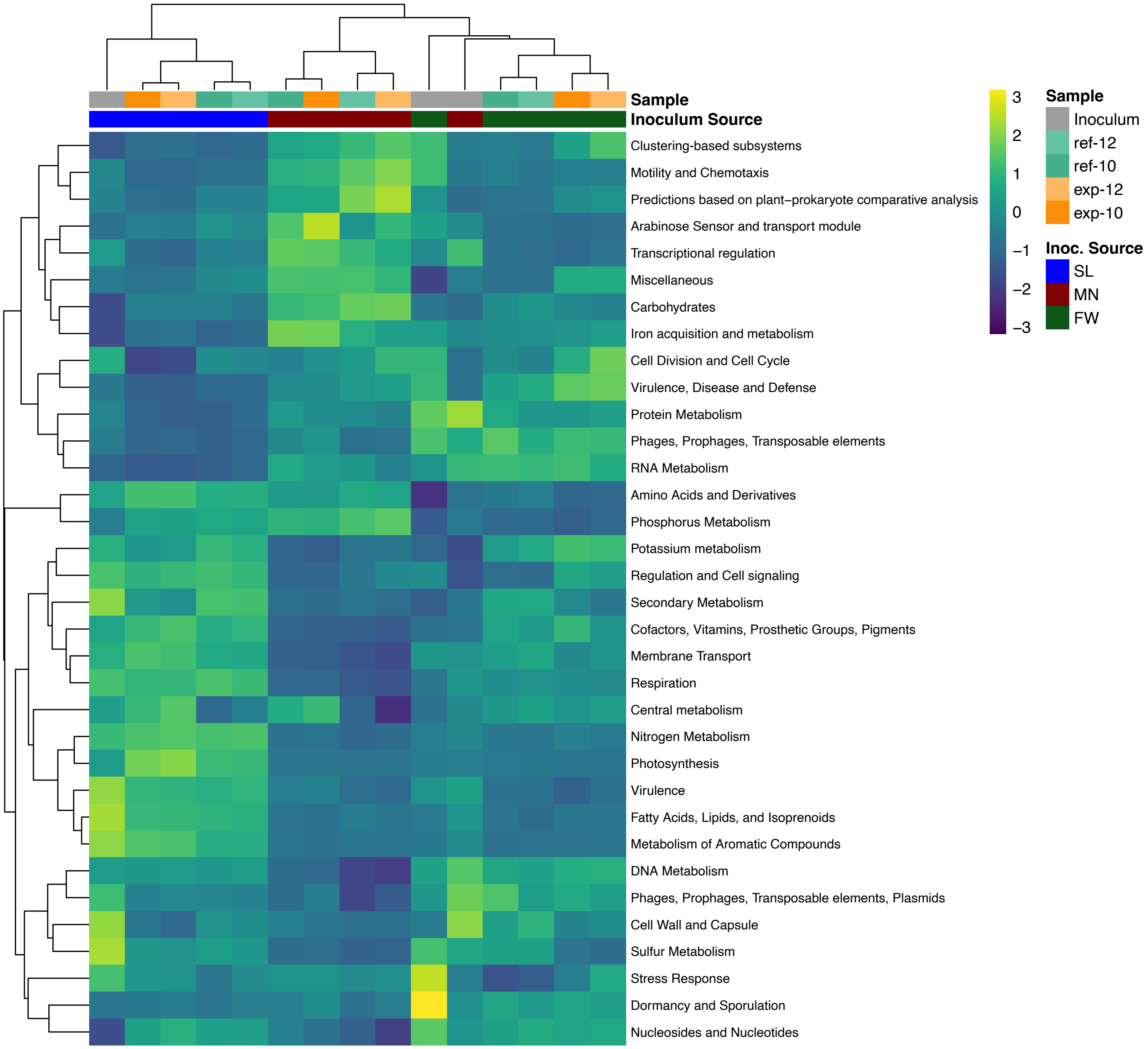

Figure S8. Row-scaled heatmap visualizing the functional composition of the reactor samples at SEED subsystem level 1. Samples are are either reference (ref), experimental (exp), or corresponding inocula, optionally suffixed with weeks since inoculation.

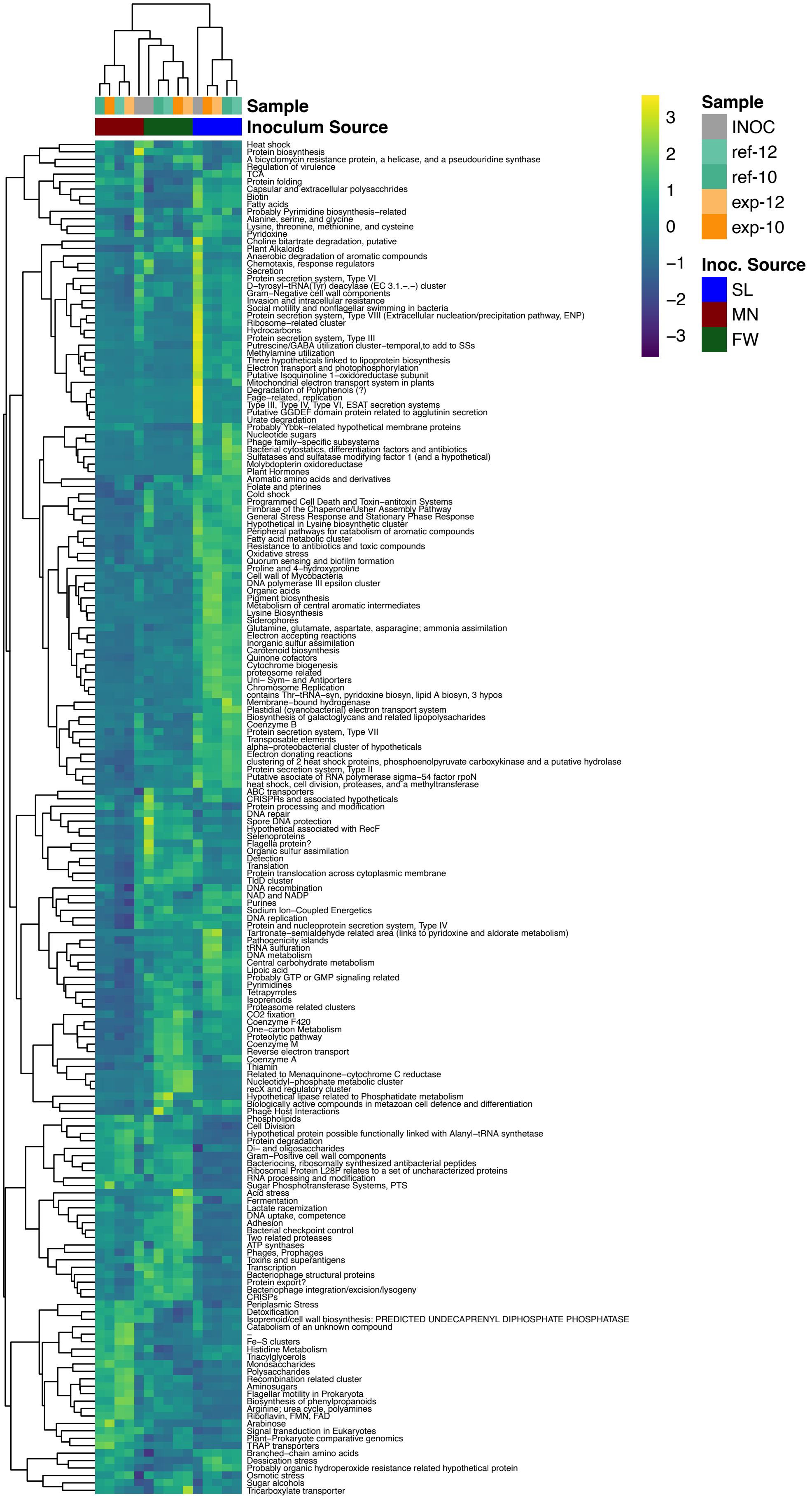
Sample
Inoculum Source
3
2
1
0
−1
−2
−3
Sample
INOC
ref-12
ref-10
exp-12
exp-10
Inoc. Source
SL
MN
FW
Heat shock
Protein biosynthesis
A bicyclomycin resistance protein, a helicase, and a pseudouridine synthase
Regulation of virulence
TCA
Protein folding
Capsular and extracellular polysacchrides
Biotin
Fatty acids
Probably Pyrimidine biosynthesis−related
Alanine, serine, and glycine
Lysine, threonine, methionine, and cysteine
Pyridoxine
Choline bitartrate degradation, putative
Plant Alkaloids
Anaerobic degradation of aromatic compounds
Chemotaxis, response regulators
Secretion
Protein secretion system, Type VI
D−tyrosyl−tRNA(Tyr) deacylase (EC 3.1.−.−) cluster
Gram−Negative cell wall components
Invasion and intracellular resistance
Social motility and nonflagellar swimming in bacteria
Protein secretion system, Type VIII (Extracellular nucleation/precipitation pathway, ENP)
Ribosome−related cluster
Hydrocarbons
Protein secretion system, Type III
Putrescine/GABA utilization cluster−temporal,to add to SSs
Methylamine utilization
Three hypotheticals linked to lipoprotein biosynthesis
Electron transport and photophosphorylation
Putative Isoquinoline 1−oxidoreductase subunit
Mitochondrial electron transport system in plants
Degradation of Polyphenols (?)
Fage−related, replication
Type III, Type IV, Type VI, ESAT secretion systems
Putative GGDEF domain protein related to agglutinin secretion
Urate degradation
Probably Ybbk−related hypothetical membrane proteins
Nucleotide sugars
Phage family−specific subsystems
Bacterial cytostatics, differentiation factors and antibiotics
Sulfatases and sulfatase modifying factor 1 (and a hypothetical)
Molybdopterin oxidoreductase
Plant Hormones
Aromatic amino acids and derivatives
Folate and pterines
Cold shock
Programmed Cell Death and Toxin−antitoxin Systems
Fimbriae of the Chaperone/Usher Assembly Pathway
General Stress Response and Stationary Phase Response
Hypothetical in Lysine biosynthetic cluster
Peripheral pathways for catabolism of aromatic compounds
Fatty acid metabolic cluster
Resistance to antibiotics and toxic compounds
Oxidative stress
Quorum sensing and biofilm formation
Proline and 4−hydroxyproline
Cell wall of Mycobacteria
DNA polymerase III epsilon cluster
Organic acids
Pigment biosynthesis
Metabolism of central aromatic intermediates
Lysine Biosynthesis
Siderophores
Glutamine, glutamate, aspartate, asparagine; ammonia assimilation
Electron accepting reactions
Inorganic sulfur assimilation
Carotenoid biosynthesis
Quinone cofactors
Cytochrome biogenesis
proteosome related
Uni− Sym− and Antiporters
Chromosome Replication
contains Thr−tRNA−syn, pyridoxine biosyn, lipid A biosyn, 3 hypos
Membrane−bound hydrogenase
Plastidial (cyanobacterial) electron transport system
Biosynthesis of galactoglycans and related lipopolysacharides
Coenzyme B
Protein secretion system, Type VII
Transposable elements
alpha−proteobacterial cluster of hypotheticals
Electron donating reactions
clustering of 2 heat shock proteins, phosphoenolpyruvate carboxykinase and a putative hydrolase
Protein secretion system, Type II
Putative asociate of RNA polymerase sigma−54 factor rpoN
heat shock, cell division, proteases, and a methyltransferase
ABC transporters
CRISPRs and associated hypotheticals
Protein processing and modification
DNA repair
Spore DNA protection
Hypothetical associated with RecF
Selenoproteins
Flagella protein?
Organic sulfur assimilation
Detection
Translation
Protein translocation across cytoplasmic membrane
TldD cluster
DNA recombination
NAD and NADP
Purines
Sodium Ion−Coupled Energetics
DNA replication
Protein and nucleoprotein secretion system, Type IV
Tartronate−semialdehyde related area (links to pyridoxine and aldorate metabolism)
Pathogenicity islands
tRNA sulfuration
DNA metabolism
Central carbohydrate metabolism
Lipoic acid
Probably GTP or GMP signaling related
Pyrimidines
Tetrapyrroles
Isoprenoids
Proteasome related clusters
CO2 fixation
Coenzyme F420
One−carbon Metabolism
Proteolytic pathway
Coenzyme M
Reverse electron transport
Coenzyme A
Thiamin
Related to Menaquinone−cytochrome C reductase
Nucleotidyl−phosphate metabolic cluster
recX and regulatory cluster
Hypothetical lipase related to Phosphatidate metabolism
Biologically active compounds in metazoan cell defence and differentiation
Phage Host Interactions
Phospholipids
Cell Division
Hypothetical protein possible functionally linked with Alanyl−tRNA synthetase
Protein degradation
Di− and oligosaccharides
Gram−Positive cell wall components
Bacteriocins, ribosomally synthesized antibacterial peptides
Ribosomal Protein L28P relates to a set of uncharacterized proteins
RNA processing and modification
Sugar Phosphotransferase Systems, PTS
Acid stress
Fermentation
Lactate racemization
DNA uptake, competence
Adhesion
Bacterial checkpoint control
Two related proteases
ATP synthases
Phages, Prophages
Toxins and superantigens
Transcription
Bacteriophage structural proteins
Protein export?
Bacteriophage integration/excision/lysogeny
CRISPs
Periplasmic Stress
Detoxification
Isoprenoid/cell wall biosynthesis: PREDICTED UNDECAPRENYL DIPHOSPHATE PHOSPHATASE
Catabolism of an unknown compound
−
Fe−S clusters
Histidine Metabolism
Triacylglycerols
Monosaccharides
Polysaccharides
Recombination related cluster
Aminosugars
Flagellar motility in Prokaryota
Biosynthesis of phenylpropanoids
Arginine; urea cycle, polyamines
Riboflavin, FMN, FAD
Arabinose
Signal transduction in Eukaryotes
Plant−Prokaryote comparative genomics
TRAP transporters
Branched−chain amino acids
Dessication stress
Probably organic hydroperoxide resistance related hypothetical protein
Osmotic stress
Sugar alcohols
Tricarboxylate transporter

Figure S9. Row-scaled heatmap visualizing the functional composition of the reactor samples at SEED subsystem level 2. Samples are are either reference (ref), experimental (exp), or corresponding inocula, optionally suffixed with weeks since inoculation.

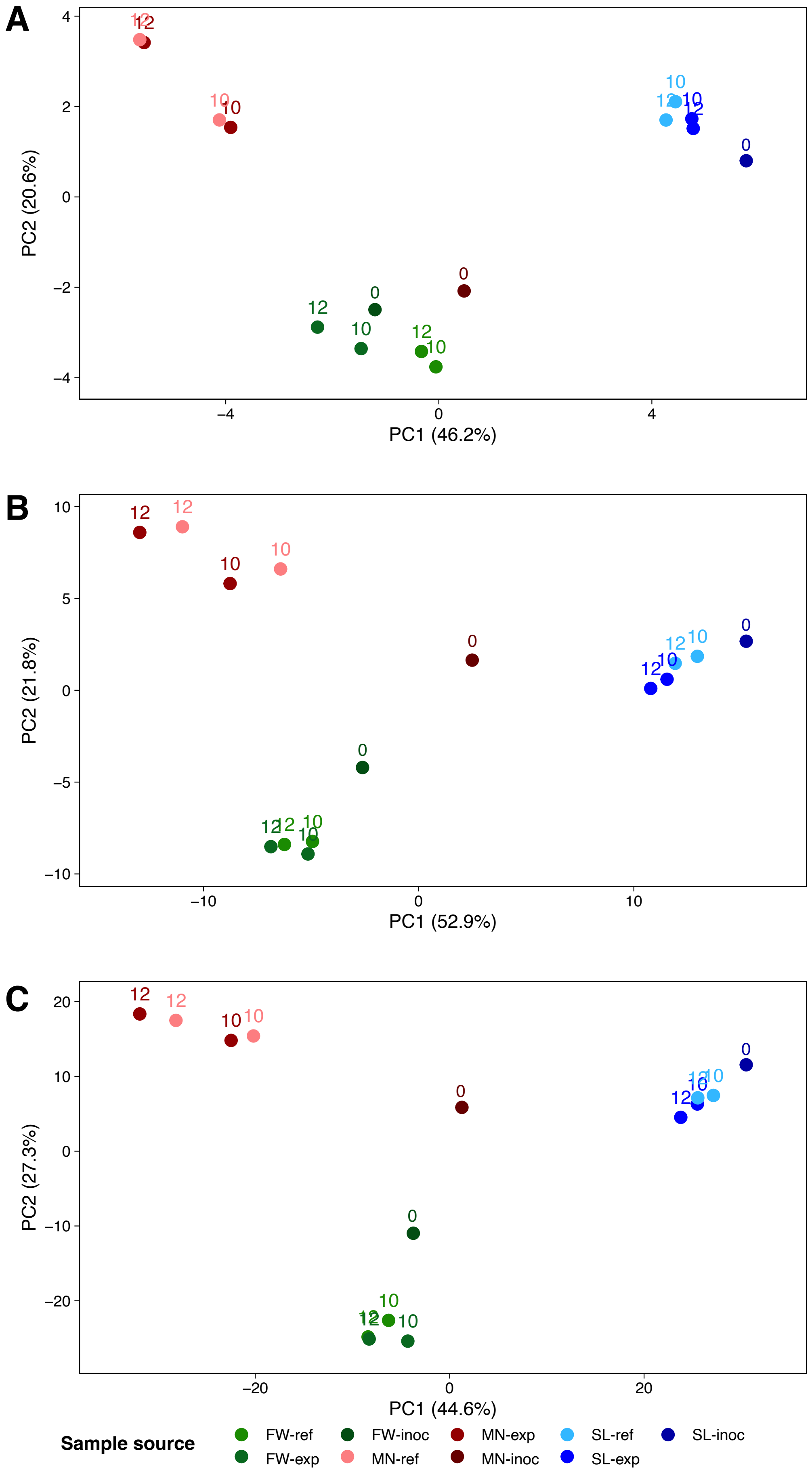
A
B
C
PC1 (46.2%)
PC2 (20.6%)
PC1 (52.9%)
PC2 (21.8%)
PC1 (44.6%)
PC2 (27.3%)
Sample source
FW-ref
FW-exp
FW-inoc
MN-ref
MN-exp
MN-inoc
SL-ref
SL-exp
SL-inoc

Figure S10. Principal Component Analysis (PCA) plots of functional composition at SEED levels 1 **(A)**, 2 **(B)**, and 3 **(C)**. Numbers indicate weeks since inoculation.

Table S1: Adjusted p values (Holm method) for the all-pairwise multivariate comparisons of experimental reactor operational parameters.
* $p < 0.05$

	SL-exp	MN-exp	FW-exp
SL-exp			
MN-exp	0.476		
FW-exp	0.476	0.033*	

Table S2: Adjusted p values (Holm method) for the multivariate comparison within each reactor pair (i.e., comparing reference and experimental reactors for each inoculum source).

**Reactor pair**	***p* value**
SL	0.794
MN	0.794
FW	0.096
