# Supplementary material for: Converge or Diverge? Exploring the Fate of Taxonomically Different Anaerobic Digestion Communities Under Uniform Growth Conditions
Source: Microb Biotechnol. 2025 Sep 24;18(9):e70233. doi: 10.1111/1751-7915.70233 (PMC12457802; doi:10.1111/1751-7915.70233)
Supplement: Supplementary file 2 — File S2: Krona plot of microbial community composition in inocula and reactors. [file MBT2-18-e70233-s001.html]

Javascript must be enabled to view this page.

magnitude
magnitudeUnassigned

FW-ref-10
FW-ref-12
SL-exp-10
SL-exp-12
SL-ref-10
SL-ref-12
FW-inoc
MN-exp-10
MN-exp-12
FW-exp-10
FW-exp-12
SL-inoc
MN-inoc
MN-ref-10
MN-ref-12

2287591247793235653274956453209678621863731459754346391569627813068941329401514946711532900840571945875477

1006618105077774506012411286344696588013674558697998666117767094793926640268325581616103136
000000000000000

375534242987175234413811761613237779335751
0000000000

00000
454102132233301881

00000
454102132233301881

00000
454102132233301881

454102132233301881
00000

45411661881

354

4545673221810

0000000000
330134241966143034413811428613049679335751

0000000000
330134241966143034413811428613049679335751

0000000000
330134241966143034413811428613049679335751

0
13884

9784

4100

0000000000
330134241966143034413811294910690079335751

330134241966143034413811294910419479335751

2706

1337
0

1337

0
9712

2272

2790

4650

000000000000000
99705710400927253191213531572116607407366971571016760011692749402862489245093214961564546

000000000000000
996746103917243720272660828077431207136677140611536471166248936591404114449333905756864

996746103917243720272660828077431207136677140611536471166248936591404114449333905756864
000000000000000

98819910294764114957074572486522896783641744061153647115798693151684774364133846356864
000000000000000

30433231118632102437
00000

30433231118632102437

0
300

300

000000000000000
98295310236324107137062652479802890323621664061153647115234692729484774364133846356864

203012067619903027155016087455134120412166016052250975112491948

2531725739749911697562266109152105613422692519375116039481429

457044619285061364360006812171961586199847422355902801802015652264

206454229925589989314533527571872591775672982982835337115459348813

26081271816921118054932561198412562354029094200611642915712252

80432815112286264222791078111257503072733814477850096228237214288033144618

4375244654100451559880928549166201822232646925339787492125517802757

3044333134803083247800240912881411382613753184143461142747497771397071285519444

3752937923107291769473908865140971408181541021302472591641714542097

28597286911166418422948410752103831206153830213219335271473012391673

7209073917544618235444876524142645327283482789085730113213069626713981

429934341223965377861623019206162961676218846169332105932017417112543

1877019145543086404035503771167651068205001466587877361067

30500311864146658933343729111321410185134088243181481014361978

000000000
22032313782119267264682224301785

22032313782119267264682224301785

0000
440438408371

440438408371
0000

440438408371

41814356347318151342822865
0000000

0000000
41814356347318151342822865

27322860347318103229732198

282340

747730286868667

420426195441

2811166693923432245214457
000000

000000
2811166693923432245214457

2811166693923432245214457

16772352111748001237041655320212779378
000000000

7087649734800118033131475036184
00000000

21081863315122061491

29029823872048397026701912

8747801241866583

438335264

41846643653310923370705031934

96915881440567134062027743194
00000000

452704487449

463886569528

525837582539

290291360

716494443

383410428319202402231

282731542411

651413359

296329358372

276234

286717584259307080998520594
0000000

0000000
286717584259307080998520594

352825

281

286717583907307069938520594

000000
2249226938088222951839

000000
2249226938088222951839

2249226938088222951839

31192028811748692329134229533620016490139533026369520851364388105587682
000000000000000

220634374395245832232742428625702064814799023691108
0000000000

0000000000
220634374395245832232742428625702064814799023691108

0000000000
5920444233215709163545376125701938884044918141108

42827264821672361252527517941508287632559

53569533624056121522177662591652712551108

142427321432144053112205953

909548793924430057504339964991

5026071557118445315855346

739297236
000

739297236

00000000
1614303294232982669197525123577541555

1614303294232982669197525123577541555

000000000000000
31192067483112528455106259420012204113833026369520321639881896574

31192067483112528455106259420012204113833026369520321639881896574
000000000000000

0000000000000
59167483112528455106259412204113832518330111901639881896574

13215161211428915230767

5911201243587527875201220411383251833011639881896574

37215465752174034242

1462176412691692423

3579448129343910

000000
311329200508394842

311329200508394

368

474

00
15274358

00
15274358

00
15274358

15274358
00

15274358
00

15274358

000000000000000
956110685159862417339811325264842643727255686932957958415572406832839

10127551019
000

000
10127551019

000
10127551019

10127551019
000

10127551019

000000000000000
956110685159862417338799317714842643727255686932956939415572406832839

956110685159862417338799317714842643727255686932956939415572406832839
000000000000000

0000000000
101608579312072734626437272552030352242406832839

10160857931207273462030
00000

9592857929472257442030

56811801081

555521

00000
2643727255352242406832839

2536026225352242314931586

107710309191253

582610112759244254909
00000

00000
582610112759244254909

31065393405223272412

27204719354020982497

9561106855482484686932956333
0000000

0
273

273

000000
956110412484686932956333

88089549484627632956333

753863593

0
5482

3533

1949

000
19555171161904

19555171161904
000

000
19555171161904

19555171161904
000

000
19555171161904

19555171161904

000000000000000
1280973142715528202673715325146231715275721092299337693668641151891271234607612282691464575339755785772341

00000000000
477683497465506311143948283621320752527262

477683497465506311143948283621320752527262
00000000000

00000000000
477683497465506311143948283621320752527262

00
449322

322
0

322

0
449

269

180

477683497465506311143943793621320749307262
00000000000

0
193

193

00000000000
477683497465506311143941863621320749307262

598334341114394

3127

430

301

47768349767362132072867262

373

413

1482862

186

543507328
000

000
543507328

000
543507328

000
543507328

000
543507328

543507328

000000000000000
1124482402242418867420233026534586388425161285787641857231852037782181

00000000000000
37490416620131961489449261552176971685052582242223821380

5946224381530
0000

5946224381530
0000

00
308302

308302

236
0

236

286239
00

286

239

438
0

438

00
320504

272

232

320

551
0

551

0000
3565149441221

197944261
000

000
197944261

944

261

197

000
356317649

0
649

234

415

356
0

356

0
317

317

0
311

0
311

311

00000
3046384764839485432

3046384764839485432
00000

3046384764839485432
00000

35264990

331

20492986

304638479081178432

000000
5995277339013914105032718

4281435
00

0
1187

256

317

370

244

428248
00

250248

178

0
322

0
322

322

00000
4723183252635433

0
1121

1121

0
942

942

00
263432

263

432

0000
4723183251478

341

4723183251137

0
419

419

225
0

225

0
816

320

496

16645921023108911298
00000

16645921023108911298
00000

5283693473250

317

11365926547426702

1029

0000
3795053591548

359347
00

359

347

305
0

305

379505669
000

322

379505

347

227
0

227

0
444

444
0

444

402
0

0
402

402

483
0

0
483

232

251

2370902132410966616
00000

2370902132410966616
00000

2370902132410966616

4365
0

563
0

563

3802
0

229

1173

2400

00000
14891279857899372

00000
14891279857899372

10161279421470372

473436429

251
0

251
0

251
0

251

0
465

0
465

0
465

233

232

388118601166
000

388118601166
000

11332
0

554

10778

246
0

246

388531
00

388531

0
635

635

282
0

282

0
374

0
374

374
0

374

186911647168502422
00000

186911647168502422
00000

00000
186911647168502422

186911647168502422

18628735904570
0000

333
0

0
333

333

18628735904237
0000

0
3370

359

471

359

2181

0
191

191

00
1862873

1167527

695346

201
0

201

0
198

198

308
0

308

0
243

243

0
316

316

000
82512161380

000
82512161380

000
82512161380

82512161380

11244786613383122472882715371296602332747121417914133141160981396801
000000000000000

0
260

0
260

260
0

260

291476
00

00
291476

291476
00

291476

656
0

0
656

0
656

656

000000000
2874063161912238436126571157116319688

253
0

253
0

253

0
1144

1144
0

1144

196
0

196
0

196

2872050648106073236863519306319688
000000000

0
302

302

00
307840

307840

9091903
00

5781903

331

253
0

253

312296369
000

312296369

0000
287436443523

287436443523

0
614

614

0
688

688

0
558

558

00
3151033

3151033

14867
0

889

474

2798

472

355

240

441

8514

684

0
999

691

308

000
360391393

360391393

0
302

302

4862
0

4862

00
4221630

495

422310

235

227

363

1254389473
000

399389473

855

479
0

479

000
325315366

325315366

0
241

241

0
1262

415

549

298

9296487275306587
00000

9296487275306587

555
0

228

327

0
374

374

5621557
00

5288557

333

0
433

433

0
369

369

0
340

340

000
4493374416

4493374416

0
4136

4136

000
6423948196

575

501

289

6423946831

0
670

269

401

20139711631112520163272
000000

0
201

201

449
0

449

0
14096

1257

12839

262
0

262

00000
20139711252112537382

897

599

6444594696476

542

136997179365618221

1645

558

4830

956

261

409

531

1457

37911083
00

610

2937

530

496

665

252

345

523

252

304

656

3791034

1465

249

468

297

0
509

509
0

509

245
0

0
245

0
245

245

00
220234

00
220234

220
0

220

234
0

234

457470242
000

000
457470242

000
457470242

457470242

000000
9041021123517902178457

904102194512912178016
000000

226
0

226

000
3077431163

280

326372

307417511

566
0

338

228

217
0

217

00000
90410216385484738

459

467

659

90410216385481163

841

426

253

470

1098
0

1098

0
225

225

290499441
000

0
290

290

499
0

499

0
441

441

3418232154220763715999526162953
000000000

0
1659

277
0

277

0
195

195

0
183

183

0
511

511

0
294

294

199
0

199

20745992953
000

0
1544

217

247

244

386

255

195

315
0

315

2155992953
000

5992953

215

0000000
341823215422071190952347

21542207
00

11151478

442

597729

271
0

271

000
341456535

341456535

0
347

347

0
186

186

262
0

262

367208417
000

367417

208

0
263

263

356
0

356
0

356

0
466

253
0

253

0
213

213

179
0

0
179

179

225269
00

225269
00

269

225

222
0

222
0

222

0
1019

0
766

766
0

766

253
0

0
253

253

00
3942164

00
3942164

3942164
00

3942164

000000000000000
1090367566262740014606789218981133974712141630665434662708801

327
0

327
0

327

00
606707

606707
00

606707

0
289

289
0

289

1057967566262740013728758718086133974711535521617461903708801
000000000000000

80634193217427614251855385932427141903
0000000000

408365471

765538282174276142538588532714

18551903

381333247257
0000

247

257

381333

00000000000000
213522304088463913303758715984954747221125021489708801

524

266

312414

520

289

2135223040884639286827491500395474722112502708801

260

197

298

98254838

0
201

201
0

201

38344702759
000

1192
0

245

479

468

38332782759
000

770

383

2034

2759

474

0
199

0
199

199

324482305
000

324482305
000

324482305

0
396

396
0

396

0
206

0
206

206

0
382

382
0

382
0

382

293
0

293
0

0
293

293

198
0

0
198

198
0

198

218
0

218
0

0
218

218

319
0

0
319

0
319

319

0000
10767214292315

10767214292315
0000

0
429

429
0

0
429

429

10767212315
000

0
539

0
539

539

10767211776
000

10767211776
000

10767211776

23405820460732168245012928486425577186983212521161696146559947292426285967279233002163711
000000000000000

416
0

416
0

197
0

0
197

197

0
219

0
219

219

39912112032
000

000
39912112032

0
778

0
261

261

517
0

237

280

000
3994332032

3994332032
000

3994332032

00
2822970

2822970
00

0
2970

2970
0

2970

282
0

0
282

282

000000000000000
23365920460732168245012928486425543586567212521161696146559947292405495962277233002163711

000000000
11191397339324551378373119115961

000000000
11191397339324551378373119115961

0
307

307

00
259356

259356

000
3636123486

3636123486

00000000
49742933932437837388410565

3782611

2503924

3393246344030

497429373

00
5511910

270

281

1910

0
2255

0
2255

2255
0

2255

00000000000
2503243053372958892007904285311821604591797

239
0

0
239

239

8233789
00

00
8233789

8233789

0
531

531
0

531

3984416
00

3984416
00

3984416

00000000000
104813415337294202101490424742579328231229

2212579
00

221

2579

4673431785
000

3431

467785

202
0

202

0
2766

2766

0
263

263

401
0

401

0
546

546

0
238

238

0000000
677134140842338719655455

387

470

4206174085885455

253

327

247

183

257397

240

407

0
2312

2312

0
371

371

195
0

195

00000
8575473197732444

3202135

5375473195597444

0000000
53332141290420872952297

53332141290420872952297

0
3348

3348

0
2903

2903

444
0

179

265

00000000
145510891226595379788817515568

11155162067
000

11155162067

00
13471947

1117

2301947

3405734372204
0000

340573

437

2204

00
2532931

2532931

0
245

245

0
5371

782

4589

000
544379917

544251

379

666

5958366568
000

2102

5956264568

1916
0

0
1916

1916

222
0

222
0

222

2889
0

0
2889

2889
0

2889

00
14293780

00
14293780

00
14293780

14293780

23003720049032064945012928296225474278063209170161696145282918762174425845252230642163711
000000000000000

0000000000
58185582855226881183478113791847684

5818551973
000

1973

581855

00000000
4695522688118325368791847684

4695522688118325368791847684

0
359

359

2252527
00

2527

225

0
3230

0
3230

3230

000
1843417665

5424
0

2673

2751

000
1843412241

1843412241

00000000
887123290434412569251417526293

887123290434412569251358919521
00000000

415490463186015442517095322

3705123619

47274244166858814154780

5434379535800

3208
0

3208

00
2463564

2463564

340
0

340

31548581313228608
00000

00000
31548581313228608

5827993359

266

3154852312575249

395
0

0
395

395

0
2489

2489
0

2489

00000000
133510193922334110786733322161184

00
2853866

2853866

00000000
83710193400281810785782259721184

397

5012725812555

837101918281564577217410647629

4243413653293

11489132024

3043555

2462665

4985225236662378
00000

4985225236662378

00
2492880

00
2492880

2880

249

00
2392708

2392708
00

2392708

00
6272375

00
3082375

3082375

319
0

319

0000
3001407226965792

3884690
00

3884690

3005305437
000

3005305437

000
2422435042

2422435042

359275
00

359275

000
55660447455

3042417599

25236339856

000
2502293168

2502293168

44767461075635896164799354868625803244161053070541389410867
00000000000000

23015320
00

2969

5566

2534

2304251

54134947670348891466828258032449783209161734
00000000000

364432444761336484582812651574686811691998

17691703194215256211315167029159225736

00000000
11079387888785817852636921216010867

365708670931010235669109659792

7429388027657752802311951075

00000000
447674694688115165243077126

732

336293

4476742700

637

69468881565226454426

000000
11141003396237081065660

11141003396237081065660
000000

11141003396237081065660

000
3812599398

00
2592131

2131

259

3207
0

3207

1915
0

1915

0
381

381

2145
0

2145

00
2792155

00
2792155

2792155

0
234

0
234

234

0000000000
16694222679318495917102675525661618417823595846693

0000000000
171502353017411185652534212588212842831942301

6366596917093942345

9601215116011276453104

540531395

489525481459

24153167241125933928113537467

1029127710681113396

779961940935586

554762649665447

496478358

5556586086594733422

629558645600

844212509583666422142212521701296427272301

344395347

12911693158515927403056

14979220326316754815246150183536530204980763904392
0000000000

579886699681317

41125742438541331388

10400714081411805910615434843536374253305444193704

8646116289621885838926463850440

318743813472318310072001

1349618589145021362147546764783676

1576521223168101583167055616119855688

25113538444168575396953658298666584630531417456723
0000000000000

0000000000000
2511353844416857534275324829866658463050237659723

2511353844416857534275324829866658463050237659723

5424102611
000

5424102611

2917186
00

3219

2913967

256443213512480816926818284125511174315844212347057156461555621191247178543131861
000000000000000

0000000
2063162237351713209499680

265

2063162237351710559499680

00000
287314310373725

310189315

184

410

287314

0000000
1724164643436305526416962

28914402492697

1435164629036305523924265

000000000000000
1341817088118211160579113757857320066751016136093889433112395801886118316

351047894460589221911533536851885841885824115290975722

4035546010468014338340192701687870970166767002

6184786332972222145477893

5873683928873441186814011977582492761898203438472093951788617594

000000000000000
29753839912113952446913651477571112435497791121868788150422107352

19352534524469901146835111243549779588859011149346106588

10401305912113946492253397771076764

724092485685755040122603208934932066927124184446641885806193
000000000000000

3804503923094426626506366

663779789106311328914268685719

458652458

10391218245814429818214741196

3364985943015382711

7751021979130452435825145216446383569

27132459278733232175178031307803

22692743114914665794785242227206648557915792682252304194

430597266

95511281678233675256732212612432

0
293

293
0

293

0000000
35559758017371468309171634

0000
1090539156941062

5394175

4884012466

6027507596

00
2473210

3210

247

0000000
3555975806476828402572

3555975806474456493572

2371909

3611
0

3611

2842
0

0
2842

2842

295
0

0
295

295

0000000
377205019025522927321011398

00
3772058

3772058

00000
205019022493521011398

205019022493521011398

5522280
00

3182280

234

3491410037533106889020202130119911
00000000

000000
1334436305594482196905499

1334436305594482196905499

0
242

242

2102414428
000

2102414428

349179
00

349179

7298903220
000

2643563220

197

284

268250

75612284744084064806764
0000000

75612284744084064806764

0
254

254

748
0

435
0

435

0
313

313

51862711856510
0000

51862711856510
0000

5188462510

274

3533394

4376765393807
0000

437676539740
0000

437676539740

3067
0

3067

00
271398

00
271398

271398

0000
30020944551956

347
0

347

000
3002093858

3002091146

1756

956

00
2501956

2501956

514788449138111699
00000

51454544911139278
00000

1960

5143194494825026

2266312292

2432682421
000

243268

2421

381671071686324247868566510822110310265955032026
00000000000

415
0

415

0
271

271

000000
85213662812452447719578

343497

3734337366339

342

241

2447

8314525578

303

294

2821420

316

733

2733165

4796172049

278

263

192261

191
0

191

6241521168632496596941155827424663
000000000

351646328329317

307

214

7743072236

285205

333379492

218

305

461

135241796960

2454651904

315

278

230

2733374432135

400374

3611876

358

228511

6131889

6942732035663

316459294

1033389

199

269

274

3093243214482640

247

520

336

235

4042343727

454441

3802022

7271574108853853
0000

727157484253853

2460

316181604237253507303450543922026
00000000

2582970

3655

316181604237253249301834143922026

9539

1613233078713781383173017299448
00000000

467640

213

300

1942

13781383448

475526

5362304

1893

377

302

1851904

1914786

671864243

2197

2273

272

198

423
0

423

2841575241222401829199497221972
00000000

4760
0

2609

2151

2905492516
000

2905492516

3843331994762295
00000

3843331994762295

5481923
00

2861923

262

47564382170411875802
000000

432335405

4756433893697825802

0
895

617

278

000000
5799313753827472687

5799313753824512687

296

2845218383704105701989
0000000

2845218383704105701989

00000000000000
19556515207461262940433841154051468313640381092973817279140549522

00000000000000
19556515207461262940433841154051468313640381092973817279140549522

2110

148011904541919185011586683233124319411299

19408515088461262940433387134861283313524580424650783926121138223

0000000
5004843691146425459015238

0
230

0
230

230

5254252304430
0000

0
197

197

0
230

230

00
4254430

4254430

0
328

328

2422380
00

00
2422380

2422380

000000
50048436937941308428

2559
0

2559

3092
0

3092

0
185

185

000000
50048436919441302777

194

50048436941302777

290350918541407616443563
0000000

0
5648

0
5648

5648

0000
29061235342874

0
2874

2874

0
299

299

0
1845

605

818

422

290365606
000

290348

365258

0
784

784

247
0

247

5417921563
000

000
5417921563

5417921563

000
350306542

350542
00

350542

306
0

306

00
336586

00
336586

00
336586

00
336586

336309

277

43944664518812844056045884321524905066150655651376513036823826395834724776
000000000000000

000000000000000
43944664518812844056045884321524905066150655651376513036823826395834724776

000000000000000
43944664518812844056045884269124870596141753651376512678626026352974717398

43944626912487059614175365137657267626026342024715770
00000000000

880728
00

880728

31796771143627065582
0000

31796771143627065582

39145996834130373913
0000

964224513835915130

3129793836266223

23159584122592346356

3215882033956204

43944626912416118596407665137651774626025566294575547
00000000000

43944626912416118596407665137651774626025566294575547

5493
0

5493

0000000
64518812844056045884541110951628

64518812844056045884541110951628
0000000

10071147577655

62143783223904844166541110951628

587767438491

7811048497572

0000000
52434478902358197842867378

52434478902358197842867378
0000000

28834478902358197842867378
0000000

3581978

288

3447890242867378

236
0

236

361349
00

00
361349

00
361349

00
361349

00
361349

361349

0
321

0
321

321
0

0
321

321
0

321

000000000000000
651407138799041451941246239504485699487279673857331498720417556081722

00000000
36142850165944502322466554

000
3265594232

2875594232
000

00
1391232

1391232

00
1189594

1189594

295
0

295

390
0

0
390

390

000000
36142817514502466554

0
301

301
0

301

11792466554
000

314554
00

314554

8652466
00

8652466

361428271450
0000

361428271450
0000

361428271450

259220859428206476811271472721191896357014713833
0000000000000

259220859428206476811214672721191896357012124833
0000000000000

0000000000000
259220859428206476811214672721191896357012124833

0000000000000
259220859428206476811214672721191896357012124833

304

259220859428206476811184272721191896357012124833

00
5682589

5682589
00

5682589
00

5682589

0
399

399
0

399
0

399
0

399

00000000000
49538554858613745777207319423134470

00000000000
4953855485868105777207319423134470

00000000000
4953855485868105777207319423134470

00000000000
4953855485868105777207319423134470

4953855485868105777207319423134470

564
0

386
0

0
183

183

0
203

203

0
178

178
0

178

57193631766784100432140338811311298148726296668257818316497130521722
000000000000000

000000000000000
15611361678494372140338866042418487219191735777814577924161722

000
321368665

321368665
000

321368665

1240993678494372140338859392418487219191735777814577924161722
000000000000000

307337114414603583963951076
00000000

627867268

307337358396395273

517593535

0000000000
31232163813893763485448135107879652

9457362

31232163813892818485448127745879652

000000
669840439400996996

669840439400996996

6213354971713717012988361110291109103889252601067215371070
000000000000000

6213354971713717012988361110291109103889252601067215371070

00
336446

336446

55632618156064707563610476652240519192636
0000000000

55632618156064503563610476652240519192636
0000000000

0000000000
151111616061127563144312464057520636

151111616061127563144312464057520636

5272059226283858011637232471
000000

2914297331934243211

1710195520702041

4809654298251952517584712471

000000
14011428538159315539201

14011428538159315539201

204
0

0
204

204

0000000000000
35475014217836567891584157898201386814026154316952699

35475014217836567891584157898201386814026154316952699
0000000000000

263537181591253949310661251582096039265125311517699
0000000000000

00000000
203026998940820819175938836699

203026998940820819175938836699

605101915912539493106635751412167212532681
00000000000

337

60563029624946296422681

236

3892667831030

159125394937702421253

0000000000
293291587111729651891910898462752

00
293189

293189

000000000
291587111729651873010898462752

291587111729651873010898462752

00
183290

00
183290

183290

000000
61910052172317639152683

61910051897317639152683
000000

61910051897317639152683

0
275

275

0
499

499
0

499
0

258
0

258

0
241

241

95229933463735171939
000000

95229933463735171939
000000

000000
95229930493735171939

66429911573732741939
000000

284299271373

439

3804472741939

0
243

243

00
2881892

288

214

445

219

454

273

287

0
297

297
0

297

461273
00

461273
00

00
461273

254273
00

00
254273

273

254

207
0

0
207

207

0000000
38151837822158777055329757

0000
52210009291695

52210009291695
0000

329
0

0
329

329

0
236

0
236

236

52210009291130
0000

0000
52210009291130

52210009291130

38151832602158677046038062
0000000

0000000
38151832602158677046038062

381518
00

381518
00

381518

1007784248716502878
00000

00000
1007784248716502878

1007784248716502878

22531374428329535184
00000

00000
78363613769831616

78363613769831616

00000
1470738290719703568

945738203913682577

525868602991

000000000000000
490320274128408853251669310993136453350476011640602775112576995116645055

0
229

229
0

0
229

229
0

229

000000000000000
490320274128408853251355310584113553350476011640602734402576995116645055

466434590
000

0
272

0
272

272

434590
00

00
434590

434590

194
0

194
0

194

49032027412840885325135531058466952442476011640602724902576995009545055
000000000000000

49032027412840885325135531058466952442476011640602724902576995009545055
000000000000000

235
0

235

49032027317140744625055030957243448526456911640602724902519814651042279
000000000000000

15492246461320016797791054335551189673245627

164342723913591237229692675377416652031372210423011

234287345024230376284429236610537955562490898454414481

49043226404369046081981505100140427301217940

32084516023634163193374386623641251631543001

125218231093146543873100483801446283193

53970846962024571866470426422026

000
10751021833

10751021833

000000000
9571407805101228411910571825641943

9571407805101228411910571825641943

000
474950481

950
0

0
950

561

389

00
474481

474481
00

474481

3144094071
000

000
3144094071

000
3144094071

0
992

590

402

000
3144092813

1158

491

3144091164

0
266

266

46429396333749744941139181390822364345942456853227129305659211372373540925599
000000000000000

30459243361931881037351801532312546100760553992008
0000000000

40442920495164692241
000000

0
631

631
0

631

40442914185164692241
000000

000000
40442914185164692241

40442914185164692241

000000
128114131332103211724135

000000
128114131332103211724135

128114131332103211724135
000000

128114131332103211724135

0000000000
28774224941898071037351801377510905100122953992008

1518
0

1226
0

514

712

292
0

292

2877422494185945103735180137751090599584953992008
0000000000

000000000
242216602201716499091249902274557902

242216602201716499091249902274557902

9588726064264508738442685
000000

9588726064264508738442685

0000000000
9650808058645247621124589401511531218511104

9650808058645247621124589401511531218511104

10538286128624821593865741092646904
000000000

52238030761872749232718728

5314483052437614103863413911918904

60614666348912464214429186
000000

31382373180891509140117430

292322931680295574311756

00
19225380

00
12635380

12635380

0
659

659

422
0

422
0

422

690719410
000

000
690719410

690719410
000

410
0

196

214

00
690719

690719

5716223164655307244512713
00000000

5716223164655307244512713
00000000

00000000
5716223164655307244512713

00000000
5716223164655307244512713

5716223164655307244512713

11931117043749744941136021344318498128071298365921894438758
0000000000000

0000000000000
11931117043749744941136021344318498128071298365921894438758

0000000000000
11931117043749744941136021344318498128071298365921894438758

11931117043749744941136021344318498128071298365921894438758
0000000000000

11931117043749744941136021344318498128071298365921894438758

0000000000
27782252110173556940505341737761232262885922833

27782252110173556940505341737761232262885922833
0000000000

27782252110173556940505341737761232262885922833
0000000000

1269936622035569405056426511232262885922833
0000000000

1269936622035569405056426511232262885922833

00000
15091316479727753125

15091316479727753125

205
0

0
205

205
0

0
205

0
205

205

118651071387279195281528339240320060132060214768975932427421066905
00000000000000

4594583902155
0000

4594582155
000

0
1125

0
1125

1125

459458634
000

459458634
000

459458634

0
396

0
396

396

0
390

390
0

0
390

208

182

0000000000000
11865107138487019512322773643969664708206021476896784536575445

17152352394355
0000

17152352394355
0000

17152352394355
0000

536739394355

577786

602827

00000000
275321629929292388645897411467147234964

73701051118641949
0000

73701051118641949
0000

15212184

12531797380529

6188101111893

531778

19302948373527

507622

10101372

00000
27187342762610920164225

2727353916991350
0000

332

670880441370

8751049552561

11821610374419

0000
2361299419371379

371305

348

594812

9711199741645

796983477429

0000
513699527467

513699527467

347
0

347

0000
618823465466

618823465466

0000
1548212914321348

340328

471621356308

602879377359

475629359353

0000
8911201401398

8911201401398

0000
110213321525934

386

570684669509

532648470425

0000
1942258725201821

572391

504652373311

562706304351

8761229374406

403

494362

0000
15782175343390

7861118343390

7921057

501657764550
0000

501657764550

0000
502602410385

502602410385

0000
989011463118819081

8911084802617

742779688536

582381

733813957673

506669461

780938875699

532730536424

617895466495

9509921149850

8331106597564

456

716925473500

9151003975722

512685506439

436373

530428

504652557419

659861627500

00000
3014407518581595225

12441770752541225

12961687599553

474618507501

0000000
246821214281461114011310238566

0000
12671672645666

12671672645666

0000
78591121412331990

574597

35234533486594

677987306340

15202483363

517736305

10481236441388

642

00000
2973400517951998291

2015254117951682291

9581464316

567778275
000

567778275

0000
1521209512461144

1521209512461144

0000
13071675734665

13071675734665

4207551151234374
0000

7251005745656

1562219120081780

755950979839

1165136513911099

0000
15131196364473

10591196364473

454

2468
0

295

1045

480

648

0
238

238

000
8451042375

8451042375
000

8451042375

000000
548357200110642135443835082

26713884288732
0000

8701339403

674871288

601945

526729

329

315191
00

315

191

11381402550368
0000

465668

673734550368

483323
00

483323

802866455377
0000

802866455377

439338414192226
00000

439338414192226

14662113
00

555791

437623

474699

17092329
00

496643

12131686

00
615807

615807

31653729581921
0000

739921

9571339353

470642

557827581568

442

530371175059501279
00000

473602

476

22933892412

598802

924

14631821505538355

00000
81261008030832973423

432588

560656351385

598275858371438423

433

719125118951150

2891839674388768093154
00000

15442385392

571688

530813

593

523765

503611

18262594485896

6891014346

756

516642

7241011

492662

7661005

686933

432693

11721439336554489

438519

471600343

517693

8281123298405485

10751282311368

20932866469584

6421027

483615

586605806

4743664510031625392

39404821985965463

732932

8711096331

525765

317
0

317

0000
3008345727281900

3008345727281900
0000

579855359322

452

1977260220821578

287

0000000
285159452533446716434581703

6691074356
000

6691074356

80131212215742904474
00000

485719

22303149356374

8131129305

4485712512182225474

0
591

591

0
600

600

453591
00

453591

13171954770785
0000

7951162297420

522792473365

0000
9131345350185

9131345350

185

2854013632823272039189229
0000000

1455155312571032229

2858021129628539189

560681

716

11961628442468

621

00
567729

567729

0
207

207

11101255864708
0000

11101255864708
0000

515612470382

595643394326

00000
5528574659874817877

12831695905879
0000

610774397396

673921508483

1129115916551398319
00000

1129115916551398319

9011191683628
0000

9011191683628

470683486558
0000

470683486558

489533467
000

489533467

7471106533495
0000

7471106533495

0000
509595674464

509595674464

0
321

321

6454964
00

00
6454964

390

4964

255

0
339

339
0

339

5933813413031800
0000

5933813413031800
0000

676751410

9191269408467

642918

14182096449568

501807

13131671446355

464622

000000
7332410302122998267402656625

24413203186313761202
00000

24413203186313761202

32054522296
000

550828

676890

7181071

483653

7781080296

00000
19522764460466301

7701231

686913460466301

496620

9371207326354
0000

9371207326354

634963335
000

634963335

0000
3757537212301528

13661923468593

23913449762935

00
5311333

628

531705

619804315334
0000

619804315334

121714085415151727
00000

121714085415151727

0000
20942217354391

439676

11921541354391

463

827963
00

827963

000000
4753822513592181265284

3440645513592181284

632863265

681907

75211059520122217
0000

10351483

23783341720810

513711

622930

595842380335

237832889121072

87481215835003987
0000

14822143576677

1970260017441448

16522337480569

17292401403507

9271298387

9881379297399

0000
643819548446

643819548446

727999
00

727999

0000
14851982470493

9031236470493

582746

00000
78551106216852759369

12371604381429

570687

6048877113042330369

7791140397427
0000

7791140397427

000
705922299

705922299

0000
13481854395408

450641

8981213395408

7501125298
000

7501125298

00000
819010591533342091971

3818451011491263883

43726081418429461088

00000
107051565517973000771

107051565517973000771

0000
9011138413421

9011138413421

000000
267295894613334666459

000000
267295894613334666459

267295798212051666215
000000

435

624

517718

267295

691

479631

215

569781

648

434

5711087304

18243047362

462601

448846

479639

8131063

501

450675

9641283244
000

530639

644

244

434

0000000
2687403729232344419511445

2687403729232344419511445
0000000

0
712

712

0
617

617

00
580613

580613

0000
15971390292323

491635

451

655755292323

000
44419511445

44419511445

00
510705

510705

0
511

511
0

511
0

511

0000000
109997815659891789902946371616425383643

0000
272433551314273312

23323308119772735
0000

551811

476723327

8651092303358

777974336

591811

555713

631781

8201180314

533717

756959

537796

8431154331

592898

8441213347376

437638

556827

9091232332

653989

620767

576699

492703

9511378

10641371337

483595

10691406351

467671

609826

738985

432

667865

544744

532901

530644

453

603861

567887

613754
00

613754

11421510
00

502

651

640859

00
511733

511733

0000
12191705450577

12191705450577

435
0

435

14451495
00

14451495
00

434754

444

567741

0000000
107004715271201772632909801616425383643

000
13411711467

13411711467

0000
24843310544735

24843310544735

86222812554511172432122871616145543643
0000000

3865155681538084084154136

1899427107336452644141138

2976043580456279891187

774823112908310393719062678780933643

00000
3523452011591361305

3523452011591361305

190725107088261397
00000

190725107088261397

00000
1737019985662881871321

372645419591378

269732478281062

5410608023482821678

5537611724932926643

0000
7722870426223393

238126109531107

505633

4836546116692286

302623852313573160064079
00000

3204396210171216335

3172398711301387487

2381285510951644232

3883498814381744541

53067083290030501210

14461902532665

21572904687835

764937336342

79499905443851231274

0000
6295878313341930

26563625609835

17092511385509

19302647340586

355446268151288251
00000

355446268151288251

402753539791478
0000

24263257560826

16012096419652

19802361285710
0000

9431069365

10371292285345

5465757112311772450
00000

5465757112311772450

126581725122022971971
00000

126581725122022971971

0000
12121490363500

12121490363500

6136779421722721570
00000

2544329811761273240

22472784630859330

13451712366589

00000
73389727232127634725

73389727232127634725

338645677201124
0000

338645677201124

00000
91159122883223643046113915

5966813013732140893

1662623702431149911141

21842640683823255

2445634575506980144863

2073624688613381482858

15081857430465

796311278180024661589

66538963162420901855

757932

431061189411324461

0000
12431861300345

0000
12431861300345

12431861300345

00000000000
9192059552457259790431124427822147689100738457

0000
9402130215071884

424055241181
000

602763

900

461636

488671

295

547655

298

552669293

592626295

526604

472

51627497507703
0000

465630

5961000

594

507376

961

487644

468664

438732

515

680888

515655

539729327

459

15512020
00

15512020
00

487608

592778

472634

8251037763580
0000

8251037763580
0000

8251037763580

91920591785720983411443652550214768998368457
00000000000

371388450449593996252124501870
000000000

241

509625

530688

371388212450

778856

367

501642

644

721818317618

472

459594

399308

534736

0000
18213251429248

753944

645

609962

459700

248

429

54853518072258128083720905776891403
0000000000

667

327

198244

548535131916129538371892577689492

488646

1136497121055349911203468457
00000000

2404

3251120

553499

346

497595

2974

811

3079

615

50154950156015256217
00000

280

557968

308505

499687

18081537125215251356

9141333

7146662009

511

302

523727286

11641012
00

450

7141012

25653343322450
0000

25653343322450

484
0

484

000000
177982454715502972232237

00
540802

540802

695881
00

695881

9721386
00

685

438

534701

0000
706597496181018

533769308

630853

716877310363

464658

437628

9201488334

569752

518721

499619

497699

436603321

8461082

494664
00

494664

28533679669232
0000

432599319

724970350

495618

666853

232

536639

24813415932992237
00000

6821067481410237

483679

13161669451582

454690
00

454690

000
12831852293

681973293

602879

961817
00

432

529817

612
0

612

6819974817561094
0000

765934
00

765934

0000
5371250721351

613394351

537637327

228931891035743
0000

388318

516651

288

555660

12181878359425

00
25123497

586684

496825

469614

453712

508662

00
716878

716878

00
477631

477631
00

477631

0000
516610353349

516610353349
0000

516610353349

24091583556957765182
00000

00000
24091583507454084256

00000
24091583507454084256

849774143715011296
00000

849774143715011296

431493386
000

431493386

621589495
000

621589495

1055809171920621342
00000

1055809171920621342

0000
505866763737

505866763737

495368926
000

495368926
000

495368926
000

495368645

281

00000
9154986167621905

00000
9154986167621905

0000
3326167621905

3172621905
000

3172621905

0
2995

2995

0
332

332

276261
00

276261
00

276

261

307237
00

307
0

307

237
0

237

0
252

252
0

0
252

0
252

252

0000000000000
28803023007610398575445188512229792107689431655782122603819724143633549

28803023007610398575445188512229792107689431655782122603819724143633549
0000000000000

0000000000000
28803023007610398575445188512229792107689431655782122603819724143633549

207
0

207
0

207

0000000000000
28803023007610398575445186442229792107689431655782122603819724143633549

00000000
164712282416158370909438656410

834630151199031284444003981

81359890559339624994652429

323740311183903
0000

323740311183903

450526
00

233526

217

0000
7555334551761

7555334551761

494732695728629648219149017122879064762447554645734545837
000000000000

494732695728629648219149017122879064762447554645734545837

4603204211068
0000

4603204211068

28096422557993477662248083148939540574611523627840987712
00000000000

28096422557993477662248083148939540574611523627840987712

47282648915353392
00000

47282648915353392

00
238465

238465
00

00
238465

00
238465

238465
00

238

465

6595232647
000

6595232242
000

0
467

467
0

0
467

467

000
659523899

659523899
000

000
659523899

659523621

278

225
0

0
225

225
0

225

323
0

0
323

323
0

323

0
328

0
328

328
0

328

405
0

405
0

405
0

0
405

405

4653147293121596116361021
000000

1402147293119414116361021
000000

0
545

0
545

0
203

203

342
0

342

857147293118828716361021
000000

000000
381147293118828716361021

000000
381147293118828716361021

4764

381147293118352316361021

0
476

476
0

476

5854
0

0
5854

0
5854

5854

00
59217999

00
59217999

59217999
00

59217999
00

59217999

660
0

0
660

660
0

302
0

302

0
358

358

00
19993821

0
913

913
0

913
0

520

393

10863821
00

10863821
00

00
8873821

3153821

572

0
199

199

20879410833535
0000

0000
20879410833535

0000
20879410833535

000
2087941083

2087941083
000

2087941083

0
3535

3535
0

3535

857
0

0
857

0
857

857
0

0
190

190

0
180

180

0
487

268

219

000
36454692581

36454692581
000

0
883

0
219

219
0

219

664
0

664
0

233

239

192

1302
0

202
0

0
202

202

0
1100

0
921

921

0
179

179

0
813

0
196

196
0

196

617
0

0
434

434

0
183

183

00
18342581

0
236

236
0

236

0
469

188
0

188

0
281

281

00
11292581

11292581
00

240

8892581

0
300

0
300

300
0

300

00
364337

00
364337

00
364337

337

364

000000000000000
374419529719160020205392386524277332012857255035706278139197376961659477564

374419529719160020205392386524277332012857255035706278139197376961659477564
000000000000000

374419529719160020205392386524277332012857255035706278139197376961659477564
000000000000000

24033244616674
0000

24033244616674
0000

24033244616674

000000
3434542146979082028

3434542146979082028
000000

3434542146979082028

00000000000
3740765292651448311867593310837750298735636578048370048263

3736555289031413356009780483
00000

3736555289031413356009780483

4213621448311867593310837750157435670048263
0000000000

4213629458772340874476157435642704446

170162274737244086304

118357156289252972918824303817

00000
234425505625252797564

234425505625252797564
00000

234425505625252797564

1278615389492843495131846668
0000000

0000000
1278615389492843495131846668

13111681462468

16262140625551630

984911568384133305131216668

0
330

330
0

330

00
5573073

0
3073

3073

557
0

557

317314141375881643085813411993423751580311163504461746
0000000000000

000000000
138310569054123883751337332446446943

000000000
138310569054123883751337332446446943

000000000
13831056905412388375998032446446943

0
235

235

00000000
1383105634383520480919243446943

1383105634383520480919243446943

0
617

330

287

0
375

375

0000
1553230928608158

69311299711931

257

860118016326227

0000
4063655914595045

35196011

54454811365045

323

0
3393

3393
0

551

288

2266

288

243022813161069810213
00000

17421757441010213
0000

72285416812873
0000

72285416812873
0000

72285416812873

0000
102090327297340

102090327297340
0000

1364

102090313657340

6885243166288
0000

0
535

535
0

535

333
0

333
0

333

6885241548
000

881
0

541

340

688524667
000

688524667

0
298

0
298

298

0
349

349
0

349

228
0

0
228

228

0
1161

0
581

581

319
0

319

261
0

261

0
370

0
370

370

0
316

0
316

316

0
572

572
0

572

332
0

332
0

332

301
0

301
0

301

261
0

261
0

261

00000
5832303248745197957

5832303248745197957
00000

00000
5832303248745197957

00000
5831608174331175451

5831608174331175451

0000
69574414022506

69574414022506

000
3063672396

3063672396
000

000
306367766

306367766
000

306367766

1630
0

0
1630

837

793

000
9637211847

9637211847
000

9637211847
000

000
9637211601

9637211601

246
0

246

82269362529582234
00000

0000
8226934211940

0
486

0
486

251

235

0000
8226934211454

430302741
000

430302741

197
0

197

392391224713
0000

392391224713

20410182234
000

505
0

0
505

505

2372234
00

2372234
00

2372234

204276
00

0
276

276

204
0

204

000
710590850

710590850
000

710590850
000

000
710590850

710590850

948721607542934808
00000

00000
948721607542934808

00000
948721564842934401

948721564842934401
00000

527731

448350523

948721399734541728

6764891419

00
427407

00
427407

427407

405295309815552803
00000

00000
405295309815552803

00000
405295309815552803

00000
405295309815552803

5906444

40529517689108803

740

317314112234104412403401925834211348447948
0000000000

0000000000
317314112234104412403401925834211348447948

6507041439
000

000
6507041439

6507041439

000
202018715509

202018715509
000

202018715509

98092921812136
0000

000
598553683

598553683

38237614982136
0000

38237614982136

534162316264050
0000

0000
534162316264050

534162316264050

000000
101112922321243482302069

101112922321243482302069
000000

4751522152352242069

3754071347

517

53612924245041142

456
0

0
456

456

294
0

0
294

294

00
3561274

00
3561274

3561274

5862208172995514639
00000

5862208172995514639
00000

5862208172995514639

0000
69165752734855

0000
69165752734855

69165752734855

69331708221091715725823021
000000

000000
69331708221091715725823021

957295222333366

4192170812250940111098

495442956

134311352048

1154329525222583554

630177414241999

31731416121104606951413425064834249
000000000

0000
223821021153711451

30529052777

1571178632554308

36231653774366

35448865038
000

35448865038

31731416121104347730393423422517760
000000000

565962688139417700

728475103113038

317314104711041787187634299737022

547221317711558
0000

547221317711558
0000

547221317711558

00
3383326

3383326
00

249
0

0
249

0
249

249

00
3383077

00
3383077

3383077
00

3383077

2438223295565992369104369836162972770360105760784
00000000000

545468
00

545468
00

545468
00

00
545468

545468

888829117786715638
00000

0
580

580
0

0
580

580

341396
00

341396
00

00
341396

341396

0000
54743386715638

54743386715638
0000

000
547433867

547433867

0
15638

9313

6325

597
0

0
597

0
597

597

00000000000
24382232955659923688504678654527276949390122784

1184914516
000

000
1184914516

374428290
000

374428290

0
308

308

000
502486226

502486226

00000
6066527215730174591349

00
725628

00
725628

725628

6066527215005168311349
00000

00000
6066527215005168311349

2671241469467768559

3395285880599063790

29743245301622902841
00000

00000
29743245301622902841

306256
00

306256

20922493171915511253
00000

330309277

14101718772760657

682775617482319

00000
8827529917391332

8827529917391332

87543891
00

71363058
00

27811230
00

27811230

00
43551828

43551828

00
994360

00
994360

994360

624473
00

00
624473

624473

00000
20481947512642191174

00000
2048194731002577871

00000
1143107116041343445

1143107116041343445

00000
90587614961234426

90587614961234426

000
20261642303

00
366317

366317

16601325303
000

16601325303

326
0

326
0

0
326

326

000
13441117263

000
13441117263

00
528456

528456

000
816661263

816661263

00
443488

443488
00

443488
00

443488

296
0

0
296

296
0

296

24382232844778877252285374874527276335090122784
00000000000

00000000000
24382232809028676435856257902547275176790122784

00000000
125452649210538701612711585344882230

27992435237016784613

1863149414959983319

23652028148110745040

20201851148310023837

190224036995271533

28832634197013486115

1254526142212719826921061

8651044373310556

29592567203813856513

30132361433236257119012230

0000000
118417061746120474573139182164

118417061746120474573139182164

00000
4949746892541437

4949746892541437

0000
4641227966894

4641227966894

00000
58862013851505236

58862013851505236

6799734134512347727427987892784
00000000

519405462

308363

874862773567727153687892784

409302346

59256479144210731572

22191116410425915788
00000

15291116226314993337

69010477201608

430372538

364305

0000
4841026981644

4841026981644

00000
31833343183112081837

31833343183112081837

00000
22982008502639283508

14581194320225281850
00000

14581194320225281850

675539385
000

675539385

84081411498611273
00000

84081411498611273

0
198

198
0

198

550682451702911
0000

000
596503227

596503227

000
734660363

734660363

5501225995571
0000

5501225995571

1100989415
000

329299

771690415

000
603326275

603326275

838515389
000

838515389

384353
00

384353

000
451309338

451309338

000
893520333

597520333

296

0
291

291
0

291

1030764241
000

515393241
000

515393241

00
515371

515371

000
1128468581

766468320
000

766468320

362261
00

362261

727213013674342
0000

0000
727176513674028

727176513674028

365314
00

365314

00
1106674

00
1106674

00
407332

407332
00

407332

699342
00

699342
00

310

389342

000000000000000
27831335480757964898635229049725477789248612177158103909911607561853871847008182160102174

000000000000
3521262287295483297242413357383473537358568634

3521262287295483297242413357383473537358568634
000000000000

316
0

316
0

316

0000000
352126228729548329722765332443

00000
104091765229452280

104091765229452280

0
642

351

291

0
483

483

54735712542145
0000

54735712542145

0
273

273

0
1896

1896

00
27617521

3982

10362

2763177

0
1030

458

273

299

0000000
3529591651540102679153698601

352151411031499757112911932

807754122511192240786669

0
309

309

258
0

258

0
302

302

328
0

328

4865901890
000

486590403

243

326

357

561

493
0

493

00000
9587074642931516

9587074642931516

0
285

285

42464692762
000

355
0

355

363
0

363

000
4249152762

4249152762

4126
0

2408

617

1101

0
710

348

362

704738
00

00
704738

704738

6312972153568634
00000

00000
6312972153568634

6312972153568634

0
533

0
533

533
0

0
533

533

6138022284215482866569696170384400235989534310095833519738653491
00000000000000

384
0

0
384

384
0

384

6138022284215482866569696166544400235989534310095833519738653491
00000000000000

6138022284215482866569696164694400235989534310095833519738653491
00000000000000

15542139400818121470400742912011421012007
0000000000

15542139400818121470400742912011421012007

594992198461548286656969612461258888989036510044151508317641484
00000000000000

3062111119315482866569696547293688943001647722373911080853

2577410228523216844409375050253900684631

31046368466896819412221673792

327857971877
0000

327857971877

185
0

185
0

185

0000000000
8865819135217124667510192921979311873671183791

8865819135217124667510192921979311873671183791
0000000000

000
744543882

238
0

238

000
744543644

744543644

0000000000
489547381887816641782131951372396421152632

00000
27723039494387989106

27723039494387989106

2123169913935166417824397461796421152632
0000000000

2123169913935166417824397461796421152632

00000
959124855691212492

00
4532105

4532105

00000
506124855689107492

87155684309492

2033772368

3032430

0000000000
322629101449895545728609755141788254743159

322629101449895545728609755141788254743159
0000000000

322629101449895545728609755141788254743159

000000000000000
20606712190543794782684623846057416055230411166551123359130256150652165571717060994258

2020010105159726471380772352250782854312860316127714657078315450
000000000000000

000000000000000
199023221597264714061764595632243331538316116641405165114503

1307
0

445

862

000
597292200

597292200

000000000000000
13932030159726471406176439563224333153831613571405165114503

13932030159726471406176439563224333153831613571405165114503

976274
00

0
472

472

00
504274

504274

00000000000
1634971776921655150610981322363306451320947

250812261976773
0000

250812261976773

6418
0

6418

395017376925511506109834796231320947
0000000000

257412946925511506109834796231320947

1098443

278

000000
387116153763983637831

387116153763983637831

0000
60202599531577

60202599531577

000
8853321874

000
8853321874

8853321874

000
124014779744

000
124014779265

124014779265

479
0

479

6298641474180814972151265956671240632
0000000000

0000000000
6298641474180814972151265956671240632

0
214

214

6298641260180814972151265956671240632
0000000000

6298641260180814972151265956671240632

0000
2791949551515

2791949551515
0000

264
0

264

272242
00

242

272

179
0

179

0000
279985551273

551

220

221

299

279

245273

0
249

249

735417061083865488911622185152035038625782345019091226
00000000000000

00000000000000
33241706108386548897082185152035038625782345019091226

00000000000000
33241706108386548897082185152035038625782345019091226

33241706108386548897082185152035038625782345019091226

00
403454

00
403454

403454

000000000000000
223181509426361875276123296645165942839229682425672755716928871354

19480133142636187527612329544195018202719856204487275528441920
00000000000000

000
9472831737

312480

635283755

502

00
258356

258356

0
401

401

0
371

371

0
288

288

97774634039899677867432635528
000000000

97774631327867432635

271989967528

0
884

884

1154167318314531911
00000

1154167318314531911

00
318825

318232

593

000
5354312126

5354312126

315141958766447381
00000

315141958766447381

000000
717446108812424151460

377

331

286

532

447

44610881242449460

374526

343579

624

60946662863012471060728587785294607
000000000

457842

56376662778812471060728587785294607

2117219018751673108713939823662635850785
0000000000

400

426

358

286376

369304

5685027

596516

549

303301

349325

184

305

349

308

480

242

296506

256

435

209

209

365

3268277446394414802250

280

277770

470

227

220

242

288

215

1363113110346461151823353530823785

345

260407

222

547

00000
638467167936432569

31046747730492125

328691594444

511

586
0

586

0000
102929546344924

471

422637

28810676344924

319779

18206633455907
0000

248

219

206

201

566348814394

268324

4523151162513

534281

00
347468

347468

0
352

352

652
0

202

450

631845
00

330549

301296

00
310765

310765

92058328303531916
00000

46930617431916

4512771087353

2682225
00

2682225

0
1184

793

391

427
0

427

0000
353387602418

353387602418

0
326

326

0000
4402861255675

4402861255675
0000

318

440286937675

0000
72015314402126

2961085
00

2961085

0000
4244464402126

4244464402126

1678149492464618123112380821999671354
0000000000

000
3215701178

3215701178

0
231

231

0
911

349

272

290

1324115253254618121004102621999671354
0000000000

1324115253254618121004102621999671354

614479552
000

614479552

00000
35434286210591052

354409

34245310591052

496
0

496

0
486

486

000000000000000
863324307914255204733206338413488759299658853619334530598145420176841155229

9283623809
000

3191098
00

3191098

0
375

375

6093622336
000

6093622336

00000
1032883312413261554

1032883312413261554
00000

1032883312413261554

11996714728757168615868176781326989441193
0000000000

0000000000
11996714728757168615868176781326989441193

11996714728757168615868176781326989441193

000000000000000
8565403814255204733206282023731393085192845635180595454486064891142035

1221
0

244

371

293

313

8565403814255204733206282022510393085192845635180595454486064891142035
000000000000000

3664703802174

23546412776171687

310607468389

235616136998150633455525912914837143881474823587

53431690826646211583091140757245

32551621046857047451458754683598822225105094915678

2383113485591000526362346957628741975835799452857768639331928

242414

2825129135525482406911378696261404659773597

440
0

440
0

440

0000000000
6987418856412399204411793217336310213493

000000000
56093265765520441179243827295013493

56093265765520441179243827295013493

0000000
137892356447447796345200

137892356447447796345200

000000
441526732567638725391942

000000
441526732567638725391942

441526732567638725391942

00000
13865314426614576

6542798
00

293835

3611200

763

00000
7325311628614576

7325311628614576

00000
6278432357717915

6278431919717915
00000

627843524717915

886

509

438
0

438

218610491026211436562
00000

339747
00

339747

00
2765538

748

524

182

279

232

2762284

260

180

493

356

1026
0

401

369

256

443520
00

443520

0
2414

1007

244

739

424

000
14677386562

14677386562

00
311396

311396

0
425

425

3663121085398414
00000

00000
3663121085398414

3663121085398414

0000000000
47433210531739915874111921464311630676921781413194

00000
1002126047313061511

1002126047313061511

00
2815435

347

478

820

449

613

281419

586

1723

000
4643653464

1880

4643651584

1075
0

1075

0
296

296

00
2571274

2571274

3651781
00

843

365938

4506419428360115874111921333710119676921781413194
0000000000

940375876

299

4412419053242615874111921333710119676921781413194

00
4111422

4111422
00

4111422

00000000000
106112503731143513462464317027258721009674

106112503731143513462464317027258721009674
00000000000

106112503731143513462464317027258721009674
00000000000

106112503731143513462464317027258721009674

000000000000000
3514990971676842909132531198042574153167696771773182359926078692132807

00
337605

337605
00

337605

437465
00

00
437465

437465

000000000000000
3318990971676842909128461198039820148827696771773182359926078692132807

486461439
000

486461439

0
290

290

312399
00

312399

392
0

392

00
360327

360

327

403454
00

454

403

0
497

497

62044117694013691396
000000

6204411108401369241

243

230650

250

181262

251
0

251

377
0

377

0
290

290

0
291

291

00000
308013261824891972932

2261

6561

5840

31999

39717810433

1499132661060971932

4902

40011671

78440385568

634295210519
0000

295

519

634

210

326589
00

326589

305
0

305

000
1942435223

1016435

655223

271

0
290

290

000
15166381966

6424101966

408228

466

000000
28396919935394164472

309219

365238

17766915365394164472

389

00000000
361749643726353548412353636555

361749643726353548412353636555

0
451

236

215

0
209

209

266
0

266

0
382

382

0
273

273

0
200

200

8942890555
000

318314

5762890241

259
0

259

484257
00

185257

299

0
389

389

58374237055940620372211
0000000

197

58374237036240620372211

0
1123

242

188

345

348

000
7711576584

452297584

3191279

415194
00

415194

00
290215

290

215

257
0

257

105054037710722243
00000

105054037710722243

00
1101609

330

568

533279

000
8701315653

540318

261563

312212

297335

000
1168332707

332

536387

320

632

2899
0

2899

717184
00

717184

00
507603

289

507314

433
0

433

000000
8463631034489481523

671

846363523

363489481

29411651134
000

29411651134

13153191636
000

318456

6973191180

300

217
0

217

484
0

484

0000
10686941040423

276

792694604423

210

226

143830353365457
0000

180573

320

295

213539

282724376

216

2371501

310771237

243

551579

305

179336

869

195

235

0000
845136325601208

845136325601

208

000
463320460

463320460

0
349

349

0
385

385

0
206

206

00
4632110

2110

273

190

208
0

208

591
0

406

185

263
0

263

0
408

408

0
303

303

473526421115412344836177178981754363702953270531066189947751875
000000000000000

5454143524

85163913742560244052447376651267

186

767282111541234483617717522978787284515980850

301236

28161721666334603143242922322613411121341875

0
413

413

336418
00

336418

00000
1103390963509656

1103390696

509656

267

880381476
000

880381476

102281287928782279783
000000

8792279783

233

320

7072762408

216

315237

00
266302

266302

000
271407874

271407874
000

271

251

407

439

184

000
915810434

189434
00

189434

00
628202

202

628

287419
00

287

419

973112246456531611403011636265645166171773736084101696194717045
0000000000000

000000
4564407323895582039

000000
4564407323895582039

4564407323895582039

938602322172282433378036876142311628624656610679
000000000000

000000
39773491293141586114335810679

21268821258121491204111210679

185178212484029859

2888225454621387

93860232213258942848968761423142513208
00000000000

93860232213258942848968761423142513208

000000000000
8793116444554111576339292784215541171232819245268153816366

23372799107910317614747795162493612922355237062891
000000000000

5448587538692752

23372799535945953944779516249364232080037062891

361129545829366587948959549867553466590340852739
000000000000

361129545829366587948959549867553466590340852739

00000000000
8083991797358782455131365467757122362829

405620746

714616245022083405346965

339

179536

4893996843206292336546768901864

319339897481

402

382417

189444

0000
523201887528

523201887528

0000000
8703841184382633331249251399

18436

8703841184382633331064891399

000000000000
1167165157336226030564045164965424186522834736

8618488115262447

1167165157327615182482945164965424133902387736

9570475437492768829195833381894646109652660481823215478555302328
000000000000000

1557646
00

00
1557646

1557646

000000
1466315510310921781877

7431611667975438
00000

458778667621438

285833354

0
499

499

615
0

615

0000
426315824341

426315824341

0000
874425465439

874425465439

00
297680

297680

2368140029741836728184941471211431585986817022
00000000000

10621104414311431585
00000

360362372554

7023793517685586

254

363458445

0
351

351

3001979183614939662600600817022
00000000

335

3005143665239

401

511

197918361493966413564211783

426

00
5951534

282400

3131134

4112961549
000

4112961549

0
366

366

270
0

270

99569131914233860
00000

4483539981482

425695

5473383191683

429
0

429

839
0

839

50977209380
000

22203041380

28774168

828
0

181

229

195

223

00
1514323

00
457323

178323

279

0
325

325

0
732

732

0
362

362
0

362

216
0

0
216

216

775729499166993710272583666
00000000

45834924946695636572583666
00000000

45834924946695636572583666

317380981374370
00000

317380981374370

0
604

604

0
912

912

211557777593210101089264177036109973926778313409755302328
000000000000000

1196577217399973926
000000

399

1196577217973926

00000
5854052503573289

3289

289

250

296405357

686
0

686

4984
0

4984

524
0

524

57041814237
000

2092

5554

570418

6591

206
0

206

0
334

334

0
391

391

201
0

201

272
0

272

251
0

251

77593260551973046109166711657155302328
0000000000

77593260551920301574166789211232

5274453510765042982328

12891087375512461175
00000

0000
5794151608387

5794151608387

3993991313859732
00000

3993991313859732

311273834443
0000

311273834443

524446458
000

000
524446458

524446458

000000000
1877824348658712818052110725762182413059

00
6111824

6111824
00

467

181784

430

251

322

17206073959634505
00000

00000
17206073959634505

13846073115634505

336844

3914495
00

3914495
00

3914495

236
0

236
0

236

1705823741178189080520473252578564
00000000

00000000
1597423339111989080520473252578564

1597423339111989080520473252578564

000
1084402662

1084402662

12851068619148859913417142883977784406467830164136173106327513
000000000000000

0
467

0
467

467

000
6262071884

207
0

207

00
6261884

1884

626

2515872701023665782394977513
0000000

5872701023665782394977513
000000

5872701023665782394977513

0
251

251

128577538972967768198721688485680411135
0000000000

262304
00

262304

000
27752288263

4381

847

2098

277

2486

3679

00
3652368

2368

365

237
0

237

367
0

367

0
225

225

0
420

420

279446401
000

279446401

309
0

309

0
11456

11456

323
0

323

0
23517

14428

9089

0000000000
507490344149176882185425617677597

8259

4453883

50749034410467688218542565535597

000
653401496

653

401496

0
407

222

185

000000
10753665015184760538

10753665015184760538

367
0

367

354412
00

354412

307
0

307

0000
411285230575

411285230575

619862599103169486791532291949
000000000

302535
00

288

302247

0
230

230

419
0

419

0
407

407

0
287

287

619862599729
0000

619862599729

0
304

304

0
255

255

364
0

364

867915
00

867915

0
238

238

6211949
00

2441949

377

263
0

263

0000000
293310162615521595160846476

310252
00

310252

00
179248

248

179

23994236
00

19654236

434

00
6082240

6082240

1601
0

514

415

239

433

0
238

238

0
241

241

371
0

371

0
345

345

00
23810485

238

388

9499

302

296

505
0

505

000
29315521595

29315521595

20011517852211051124
00000

185
0

185
0

185
0

185

00000
20011517813611051124

00000
20011517813611051124

00000
6456573688654680

255

6456572245654680

291

897

00000
13568604448451444

7363392376

6205211887451444

185

201
0

0
201

0
201

201

309
0

0
309

309
0

309
0

0
309

309

2602352003391504946176185122589315772328070156522115131620022447816569284817724230
000000000000000

000000000000000
2602352003391504946176185122589315702328070156522115131620022447816569284817724230

507
0

507
0

321
0

321

0
186

186

0
537

537
0

537
0

537

772596120869211660149045849670548872513885501933810350
0000000000000

0000000000000
772596120869211660149045849670548872513864551933810350

3848
0

1878

1970

5680
0

5680

0000000000
77259642814904584967054813601011933810350

77259642814904584967054813601011933810350

2324168
00

4168

232

4445
0

2149

2296

000
1208688518725

1208688518725

00
3608213

3602546

3123

2544

0
2095

2095
0

2095

00000
56337040317808530

00000
56337040317808530

56337040317808530
00000

56337040313238530

457

0000
949326235442

00
949326

00
949326

949326

00
235442

235442
00

235442

0000000000000
574646771124938719465219745613686633186351731273120947090

000000
268819521031200916246221

3646
0

3646

0000
65989712581624

65989712581624

0
412

412

382
0

382

202910556493392575
00000

202910556493392575

00000
1000166434653135324

00000
1000166434653135324

286748851

435780638875

732748

524435

28659818541677

279346817738

0000000000000
7213441124938717892197217081691831002072899995357

558
0

558

0000000000000
7213441124938717892197217081691825422072899995357

300

329

330

1124774123841284820

357

406

1123

219721701446567623927

205

90619991049

459512

1880

276

267

373904610

445344

367358450

969

707117040542556
0000

0
357

357

000
707412363

707412363

000
281486482

281486482

428442
00

428442

00
266381

266381

211
0

211

00
16301632

778681

852951

0
409

409

6307171339239123890219614174324475
000000000

24127852590
000

676698

425359

1008888

241676645

1515924
00

1169924

346

278717273239138673732475
0000000

27839723919951052475

372417

273504585

604513

3209681165

424

594565
00

594565

0000
7076444174324

7076444174324

52415361
00

502527

498460

15171492

1026934

16981948

000
51863205918

1028914

471461

384371

294576617

401400

757680

22412041058

851819

648598

12241079
00

12241079

0000
35230716371148

352307688603

382

567545

00
1287342

0
222

222

1065342
00

342

432

218

227

188

1250344280316201733
00000

00
16201733

16201733

442394
00

442394

273
0

273

268
0

268

0
414

414

0
227

227

231409
00

409

231

0
10461

186

4700

5575

0
629

629

35734915653945233
00000

00000
35734915653945233

0
357

357

0
5233

2011

3222

0
236

236

0
310

310

357349662394
0000

357349291394

371

847
0

847
0

216
0

216

0
242

242

0
193

193

0
196

196

00000000000000
34789838135410224086620067892213790930316123890110275403

347898399710611403307752111193790930316121981
000000000000

0000000
99710611403307776711347136

99710611403307776711347136

0
428

428

0
965

965

0
1640

1640

175236916544263534084801
0000000

626

2294

408

112636916544263532507

17266147554375229344488
0000000

17266147554375229344488

665
0

665

1972254
00

1972254

0
3302

3302

71383996126833123157810201909110275403
000000000

192
0

192

0
196

196

7138399612683312398610201909
0000000

713839961268331231020

273

2311909

217

265

204
0

204

110275403
00

110275403

0000000
635151548467076563703171061

635151548467076563703171061
0000000

000000
635151544316338641551061

635151544316338641551061

0000
4157072253088

4157072253088

3074
0

3074

00
759413

00
447413

00
447413

255

192413

0
312

0
312

312

734103772273318
0000

734103772273318
0000

0
227

227

563
0

233

330

0
3318

3318

0
748

748

0
507

205

302

2793221052
000

217

261

383

279322

191

4557154130
000

178

203

768

223

190

252

302

347

455715

213

447

582

425

000000000
8813411438867601836654437443801455

407
0

407
0

190

217

361
0

361
0

361

1014
0

298
0

298

0
510

309

201

0
206

206

77163179311026
0000

0
311

311

0
212

212

0
358

358

0
559

559

226
0

226

0
254

254

0
495

315

180

199
0

199

0000
379891495600

379638495600

253

179
0

179

404
0

404

189
0

189

179
0

179

3921861436426
0000

392513

503436426

315

204

326

0000000
665935213883950839744201799

000
1333673513

1333673513

00
279884

209

354

279321

290
0

290

991348
00

762

229348

457324543388739735981268
000000

457324543388739735981268

0
231

231

000000
4743943881173474531

4743943881173474531

350
0

350

0
290

290

0
506

232

274

0
393

393

1250439
00

0
439

439

638
0

280

358

0
183

183

429
0

429

256
0

256
0

256

00
22061886

666
0

309

357

00
15401886

609

1886

201

208

196

326

1134355
00

511355
00

511355

186
0

186

437
0

200

237

237
0

237
0

237

2851213
00

00
285972

285972

241
0

241

0000
21244384491915

00
7381915

7381915

821
0

333

309

179

000
565438449

289438449

276

270455
00

00
270455

270

455

694
0

0
694

247

188

259

0
941

208
0

208

0
328

328

405
0

405

0
213

0
213

213

00
3222119

0
216

216

0
282

282

0
322

322

0
306

306

1315
0

544

309

462

513
0

513
0

251

262

0
239

239
0

239

0
773

0
773

773

3793293
00

0
296

296

379179
00

379

179

0
237

237

0
818

213

263

342

855
0

184

347

324

0
658

182

476

250
0

250

7192712519400470
00000

719271705400470
00000

719271276

429400470

0
187

187

389
0

389

0
545

545

289
0

289

0
404

404

000
2164052226

0
216

216
0

216

00
4052226

0
405

405

2226
0

2226

239386188257480136609192416624912278332711442742021387
00000000000

00000000000
2824194612634518113122711400327605873223756

00
3933782

3933782

5564783027844804037700573
00000000

5564783027844804037700573

0000
3815413278947

2661

2174

3815413274112

82544321044587561612504465
00000000

2694456440465

5564432108756166064

1443102537023551131916381276542185756
0000000000

84056637015331131542381172211338756

60345982237410433847

23656218631146750209179316397812138353840979631
0000000000

0000000
7545991915125003946953

7545991915125003946953

00
4036741

4036741

23580818571246559117679316347812098940146979631
0000000000

23580818571246559117679316347812098940146979631

710369
00

461369
00

00
461369

223

369

238

249
0

249
0

249

000
7543902565

000
7543902565

7543902565
000

7543902565

000
7355086116

000
4855086116

0
2775

2775

000
4855083341

2755083341

210

0
250

250
0

250

0
361

0
361

0
361

0
361

361

0
339

339
0

339
0

339
0

339

0
243

0
243

0
243

243
0

243
0

243

000000000000000
852536153381224831921326642558542304155325291923359453566063694731790

0000000000000
8525361029181702853030120235025291923194951001314141367

000
295253678

00
295312

295312
00

295312

366
0

366
0

366

0
253

253
0

253

0
196

0
196

0
196

196

00
2383084

0
238

238
0

238

0
242

242
0

242

0
2842

0
405

405

277
0

277

0
723

723

239
0

239

0
477

477

448
0

448

273
0

273

0000000000000
8525361029181702823530120166325291923157331001314141367

00000
6417447313287838210361

0
350

350

00
314315

314315

0
266

266

641744731297383829430
00000

641744731297383829430

85253638743697149482173816632529192345681001314141367
0000000000000

85253638743697149482173816632529192345681001314141367
0000000000000

85253638743697149482173816632529192345681001314141367

270
0

270
0

270

295
0

295
0

295

239
0

239
0

239

50474078259316072085423041553149453465933553330423
00000000000

0
677

0
677

368
0

368

0
309

309

208
0

0
208

208
0

208

288709
00

00
288709

00
288709

288709

0000000000
50474078230516075423041553135593465933553330423

0
252

252
0

252

50474078230516075423041553133073465933553330423
0000000000

50474078230516075423041553133073465933553330423
0000000000

27982609136895978072825

47854230415538963437683553330423

177114699376484604

7989371505
000

000
798937317

798937317
000

00
375513

375513

000
423424317

423424317

0
1188

0
1188

0
1188

626

562

3075
0

0
3075

1759
0

0
217

217
0

217

0
546

546
0

308

238

0
597

0
597

597

0
399

0
399

399

857
0

347
0

347
0

347

510
0

252
0

252

258
0

258

200
0

200
0

0
200

200

0
259

259
0

0
259

259

000
715394447

208394447
000

208394447
000

0
208

0
208

208

394447
00

00
394447

394447

315
0

0
315

0
315

0
315

315

192
0

192
0

0
192

192
0

192

558
0

371
0

371
0

371
0

0
371

371

187
0

0
187

0
187

187
0

187

0000000000
6133925014443021997139514661545332895057

6133925014443021997139514661545332895057
0000000000

6133925014443021997139514661545332895057
0000000000

0000000000
6133925014443021997139514661545332895057

0000000000
6133925014443021997139514661545332895057

48817398824302149713956671202325523925

1252185262050079934307371132

2722313010428208143835873
0000000

0000000
2722313010428208143835873

2722313010428208143835873
0000000

8145873
00

8145873
00

8145873

272231301042820383
00000

00000
272231301042820383

272231301042820383

0
4445

0
4445

0
4445

0
4445

0
4445

4445

00
4351538

238
0

238
0

238
0

0
238

238

0
1306

1306
0

1306
0

1306
0

1306

197232
00

197232
00

197232
00

197232
00

197232

818
0

0
818

0
818

818
0

0
818

818

000000
47605176377199619901261

00000
96337745219901261

96337745219901261
00000

96337745219901261
00000

00000
96337745219901261

96337745219901261

000
476042131544

0
244

0
244

244
0

244

2869324
00

00
2869324

0
1320

947

373

1549324
00

1549324

189138891300
000

000
189138891300

000
189138891300

115322941027

7381595273

0000000
840556725981950016603169635692

322369
00

322369
00

322369
00

00
322369

322369

000
3501851762

3501762
00

340
0

0
340

340

0
258

258
0

258

3501164
00

484
0

225

259

00
350680

350680

0
185

0
185

0
185

185

840556725879150016418144125692
0000000

000000
9441281411400418329

9441281411400418329
000000

0
234

234

0
184

184

9441281411400329
00000

9441281411400329

000000
746143915838049616140835692

746143915838049616140835692
000000

000
218317121648

218317121648

3825692
00

3823678

2014

000
812529650

812529650

00000
74614391553854699311785

74614391553854699311785

356420
00

00
356420

356420
00

00
356420

356420

00
3203197

00
3203197

00
3203197

00
3203197

0
302

302

00
3202470

801

3201669

0
425

425

0000000000
25660116297191671048710565178536457554158

971671708882
0000

971671708882
0000

0000
971671708882

971671708882
0000

971671708882

00
1629313

0
439

0
439

0
439

439

00
458313

313
0

0
313

313

458
0

458
0

458

0
732

732
0

0
732

732

511
0

511
0

511
0

511
0

511

256320541364
0000

256320541364
0000

0000
256320541364

256320541364
0000

256320541364

281719690779474132583177489
00000000

000
281258242

281
0

0
281

281

00
258242

242
0

242

0
258

258

71969077947413505247
000000

000000
71969077947413505247

000000
71969077947413505247

71969077947413505247

455
0

455
0

0
455

455

0
2217

379
0

379
0

379

871
0

419
0

419

0
452

452

0
440

0
440

440

527
0

527
0

527

0000
7494110163001346

0000
7494110163001346

7494110163001346
0000

000
26703639796

812946510

18582693286

48247377300550
0000

48247377300550

000
3861426438

3861426438
000

000
3861426438

409
0

409

438
0

438

3861017
00

376

386641

7113679012998449862010
000000

0
252

0
252

0
252

252

225
0

0
225

225
0

225

352236262885092010
00000

352236262885092010
00000

2885091053
000

2885091053

35223626957
000

35223626

957

0000
359131641011844

359131641011844
0000

0000
359131641011844

359131641011844

0
181

181
0

181
0

0
181

181
0

181

00000
1192810605513232837661973

1192810605513232837661973
00000

1192810605513232837661973
00000

00000
1192810605513232837661973

1192810605513232837661973
00000

1192810605513232837661973

201
0

201
0

0
201

201
0

201
0

201
